# Oxidized low-density lipoprotein is a common risk factor for cardiovascular diseases and gastroenterological cancers via epigenomical regulation of microRNA-210

**DOI:** 10.18632/oncotarget.4152

**Published:** 2015-06-03

**Authors:** Ku-Chung Chen, Yi-Chu Liao, Jaw-Yuan Wang, Ying-Chu Lin, Chung-Ho Chen, Suh-Hang Hank Juo

**Affiliations:** ^1^ Department of Biochemistry and Molecular Cell Biology, School of Medicine, College of Medicine, Taipei Medical University, Taipei, Taiwan; ^2^ Department of Neurology, Taipei Veterans General Hospital, Taipei, Taiwan; ^3^ Department of Neurology, National Yang-Ming University School of Medicine, Taipei, Taiwan; ^4^ Graduate Institute of Clinical Medicine, College of Medicine, Kaohsiung Medical University, Kaohsiung, Taiwan; ^5^ Division of Gastroenterology and General Surgery, Department of Surgery, Kaohsiung Medical University Hospital, Kaohsiung, Taiwan; ^6^ Department of Surgery, Faculty of Medicine, College of Medicine, Kaohsiung Medical University, Kaohsiung, Taiwan; ^7^ Center for Biomarkers and Biotech Drugs, Kaohsiung Medical University, Kaohsiung, Taiwan; ^8^ School of Dentistry, College of Dental Medicine, Kaohsiung Medical University, Kaohsiung, Taiwan; ^9^ Department of Genome Medicine, Kaohsiung Medical University, Kaohsiung, Taiwan; ^10^ Department of Medical Research, Kaohsiung Medical University Hospital, Kaohsiung, Taiwan

**Keywords:** microRNA-210, SPRED2, oxLDL, DNA methylation, atherosclerosis

## Abstract

Hyperlipidemia, including the oxidized low-density lipoprotein (oxLDL) accumulation, is a risk and highly associated with the development of cancers and cardiovascular diseases. microRNA-210 (miR-210), a hypoxia-responsive microRNA regulated by HIF-1α, has been implicated in cancer and cardiovascular disease formation. Furthermore, Bioinformatics analysis revealed that the promoter of the miR-210 gene contains CpG-rich regions. It is unclear whether miR-210 expression could be epigenetically regulated in these disease progresses. The study aimed to explore the relationships between lipid and miR-210 in the context of cardiovascular disease and gastrointestinal cancer. We demonstrated oxLDL can decrease methylation in the miR-210 promoter to up-regulate miR-210. HIF-1α can bind to miR-210 promoter, but this HIF-1α binding site can be blocked by methylation. We showed that subjects of carotid atherosclerosis, stroke patients and cancer patients had hypomethylation in the miR-210 promoter, especially the HIF-1α binding site. Furthermore, miR-210 can directly inhibit sprouty-related EVH1 domain 2 (SPRED2) expressions, and SPRED2 reduces cell migration via ERK/c-Fos/MMPs pathways. Increased miR-210 and reduced SPRED2 levels were found in aorta of mice under high-fat diet and tumor tissues, which implied that miR-210 can be an underlying mechanism to explain oxLDL as a common risk factor for cardiovascular disease and gastrointestinal cancer.

## INTRODUCTION

Growing evidence has shown that oxidized low-density lipoprotein (oxLDL) is a common pathogenic factor underlying cardiovascular diseases (CVDs) and cancers [1, 2]. Accumulation of oxLDL in vascular walls was shown to be the initial culprit of atherosclerosis [3]. In addition, data showed that dyslipidemia is highly associated with the progression of multiple cancers, especially gastrointestinal cancers [4, 5]. Hyperlipidemia is also a risk factor for colorectal cancer and early metastasis of gastric cancer [6, 7]. In contrast, high concentrations of serum high-density lipoprotein (HDL), a good lipoprotein for reducing risks of CVD, were also identified to be associated with a decreased risk of colon cancer [8]. In a cohort study, an elevated serum level of oxLDL predicted future colon cancer risk [9]. In gastric cancer, variations in fatty acid levels and lipid peroxidation-related DNA adducts are associated with cancer morbidity and carcinogenesis [10, 11]. Enhanced oxidative stress was also observed in plasma samples of oral carcinoma patients [12]. Binding of oxLDL to its receptor, lectin-like oxLDL receptor-1 (LOX-1), activates multiple pathways involved in inflammation, cell migration, and angiogenesis [3, 13]. Therefore, oxLDL and its related signaling might account for the links between atherosclerotic diseases and cancers. However, more investigations are warranted to elucidate the common regulatory mechanisms of oxLDL.

Recently, epigenetic regulation has emerged as an important mechanism for several common diseases including CVDs [14] and cancer [15]. Epigenetic modifications influence genetic expressions without affecting DNA sequences. Epigenetic modifications can be dynamic processes that represent interactions among an individual's genetic background, environmental factors, and diseases. DNA methylation and microRNA (miRNA)-induced genetic repression are two major forms of epigenetic modifications. DNA methylation primarily occurs in CpG islands in the 5′ region of a gene by adding a methyl group to a cytosine ring. Three DNA methyltransferases (DNMTs), including DNMT1, DNMT3a, and DNMT3b, control DNA methylation patterns. Recent studies found that the occurrence of gastrointestinal tumors is closely related to aberrant methylation patterns at tumor suppressor genes [16, 17].

microRNAs (miRNAs) are small, endogenous, noncoding RNAs. miRNA can use sequence complementarity to bind to 3′ untranslated regions (UTRs) of messenger (m)RNAs of their target genes and cause mRNA degradation or translational repression. Several miRNAs, like miR-29b, let-7g, and miR-125, have been implicated in the regulation of oxLDL-induced signal transduction in atherosclerosis [18–20]. miR-210 was shown to be an oncogenic miRNA [21, 22] and also a proatherosclerotic factor [23, 24]. Carrying the hypoxia-inducible factor (HIF)-1α-binding sites in its promoter, miR-210 is significantly upregulated in hypoxia and can induce tumorigenesis in pancreatic, colon, and breast cancers [25]. Hypoxia has emerged as an important physiological regulator of the angiogenic switch. It has been reported that hypoxia causes angiogenesis in the progression of atherosclerosis and cancers. Hypoxia also regulates expression of miR-210. Nevertheless, there are no reports about the role of miR-210 in atherosclerosis. Several studies suggested that miR-210 expression can be regulated by hypoxia-inducible transcription factors α (HIF-α) during hypoxia. However, the detailed regulation mechanism is still not clear. The miR-210 gene is located in a CpG-rich region, but no studies have reported the epigenetic regulation of the miR-210 gene. Therefore, we aimed to investigate epigenetic regulation of miR-210 gene in the context of atherosclerosis and cancer.

In the present study, we first tested the influence of oxLDL on miR-210 expression. We next explored the effect of oxLDL on DNA methylation levels in the *miR-210* gene promoter and then tested whether DNA methylation affected the hypoxia-responsive element (HRE) in the *miR-210* promoter to block HIF-1α binding. One of miR-210 target genes, sprouty-related EVH1 domain 2 (SPRED2), was identified to explain miR-210′s effects on carotid atherosclerosis, stroke, and three cancers of the digestive tract.

## RESULTS

### DNA methylation affects miR-210 expression

**Figure 1:**
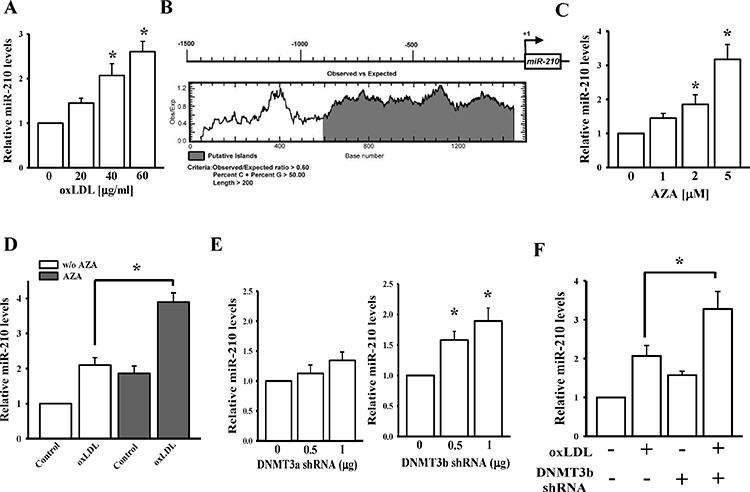
miR-210 gene regulation by oxLDL, DNMT3b, and DNA methylation **A.** The effect of oxLDL on miR-210 expression in HASMCs. After treatment with different doses of oxLDL for 48 h, miR-210 expression levels were measured by a qPCR. **B.** CpG island (gray color) prediction in the miR-210 gene promoter by CpG Island Searcher and EMBOSS Cpgplot. **C.** The effect of AZA on miR-210 expression. **D.** Co-treatment with oxLDL and AZA for 48 h on miR-210 levels. miR-210 expression levels were measured by a qPCR. **E.** Knockdown effects of DNMT3a and 3b on miR-210 levels. **F.** Co-treatment with DNMT3b-shRNA and oxLDL on miR-210 levels. Data are means ± SD of three experiments. *P < 0.05.

We first evaluated the effect of oxLDL on miR-210 expression. As shown in Figure 1A, oxLDL significantly induced a dose-dependent increase in intracellular miR-210 levels at 48 h. According to our previous study [26], oxLDL can epigenetically regulate gene expression. In addition, a previous study reported that miR-210 expression can be altered by DNA methylation [27]. To investigate whether the upregulation of miR-210 by oxLDL is due to a change in the DNA methylation level, CpG contents in the miR-210 gene promoter were first analyzed by CpG Island Searcher [28] and EMBOSS CpGplot [29]. A long CpG island (from the start point at +1 to −800) was found in this promoter (Figure 1B). Moreover, treatment of human aortic smooth muscle cells (HASMCs) with the DNA demethylating agent, 5′-aza-2′-deoxycytidine (AZA), for 48 h caused a dose-dependent increase in miR-210 levels (Figure 1C). Combined treatment with oxLDL (40 μg/ml) and AZA (2 μM) produced a synergistic effect of increasing miR-210 expression levels (Figure 1D).

We previously found that oxLDL can reduce DNMT3 but not DNMT1 [20]. To identify which type of DNMT3 can affect miR-210 expression, miR-210 levels were measured at 48 h after transfecting DNMT3a or DNMT3b shRNAs into HASMCs (Suppl. Figure 1A). As shown in Figure 1E, knockdown of DNMT3b, but not DNMT3a, significantly increased miR-210 levels. Furthermore, reduced DNMT3b levels significantly enhanced oxLDL-induced miR-210 overexpression (Figure 1F). The above data suggest that DNMT3b mediates methylation of the miR-210 gene.

### oxLDL reduces methylation of CpG islands of the miR-210 promoter

**Figure 2:**
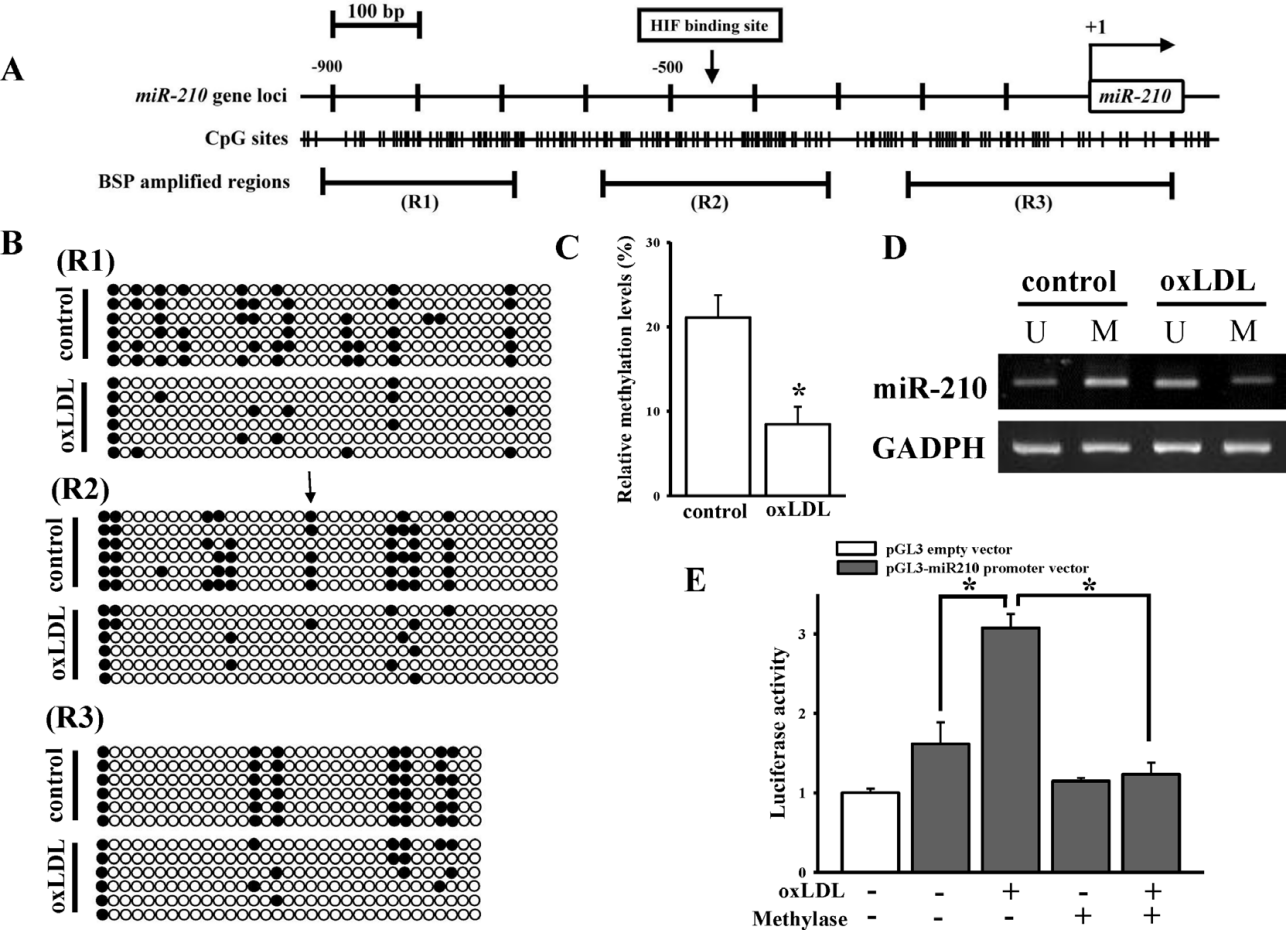
oxLDL effects on DNA demethylation in the miR-210 gene promoter of HASMCs **A.** CpG sites and the HIF-1α-binding site predictions in the miR-210 gene promoter. R1 to R3 means the regions used to detect methylation status by the BSP assay. **B.** DNA methylation changes in the miR-210 gene promoter according to the BSP assay. The HIF-1α-binding site is indicated by an arrow. Each row and circle respectively means one single sequencing reaction and one CpG site. The empty and solid circles respectively mean the un-methyl and methyl CpG site. **C.** Quantitative results for figure (B). **D.** DNA methylation changes in the miR-210 gene promoter by the MSP assay. After HASMCs were treated with 40 μg/ml oxLDL for 48 h, genomic DNA was extracted. The methylation status was determined by both the BSP and MSP assays. **E.** DNA methylation effects on oxLDL-mediated miR-210 gene promoter activity. The vector containing the miR-210 promoter was methylated by methylase *in vitro* and transfected into cells. After treatment with 40 μg/ml oxLDL for 48 h, luciferase activity was measured in triplicate experiments. Data are means ± SD of three experiments. *P < 0.05.

Bisulfite sequencing (BSP) and methylation-specific PCR (MSP) assays were conducted to investigate the effect of oxLDL on methylation of the miR-210 promoter. BSP could obtain methylation changes at every CpG residue across the whole amplicon. In the contrast, MSP could only detect the methylation status at a specific locus by using a methylation specific primer. Technically, it is difficult to obtain the full-length miR-210 promoter from bisulfite-treated DNA. Therefore, three different fragments of 200 bp from this region, named R1, R2, and R3, were amplified for analysis (Figure 2A). The R1, R2, and R3 respectively mean the region −933 to −661, −555 to −289, and −201 to 99 in miR-210 promoter. The R2 region contains a previously reported HIF-1α-binding site [25]. As shown in Figure 2B and 2C, oxLDL significantly reduced DNA methylation levels by 50% according to the BSP assay. Interestingly, a significant reduction in the DNA methylation level was found at the HIF-1α-binding site. Furthermore, a previous study [30] has reported that HIF-1α is a critical regulator to enhance miR-210 gene expression. Accordingly, we then focused on the change of methylations at the HIF-1α binding site as well as other CpG sites in the R2 region. Results of the MSP assay also implied that oxLDL significantly decreased DNA methylation levels in the miR-210 promoter (Figure 2D). An *in vitro* methylation promoter assay was conducted to further confirm that a change in the DNA methylation level influenced miR-210 promoter activity. The promoter region of 550 bp containing the HIF-1α-binding site was cloned into the PGL3 reporter vector, and then the modified vectors were treated with methylase and transfected into HASMCs. Luciferase activity was measured at 24 h after oxLDL treatment. As shown in Figure 2E, methylase treatment significantly abolished oxLDL-mediated promoter activation. oxLDL-induced demethylation of the miR-210 promoter was also found in human umbilical vein endothelial cells (HUVECs, Suppl. Figure 1B–1D). Again, methylation of the HIF-1α-binding site also significantly decreased in HUVECs.

**Figure 3:**
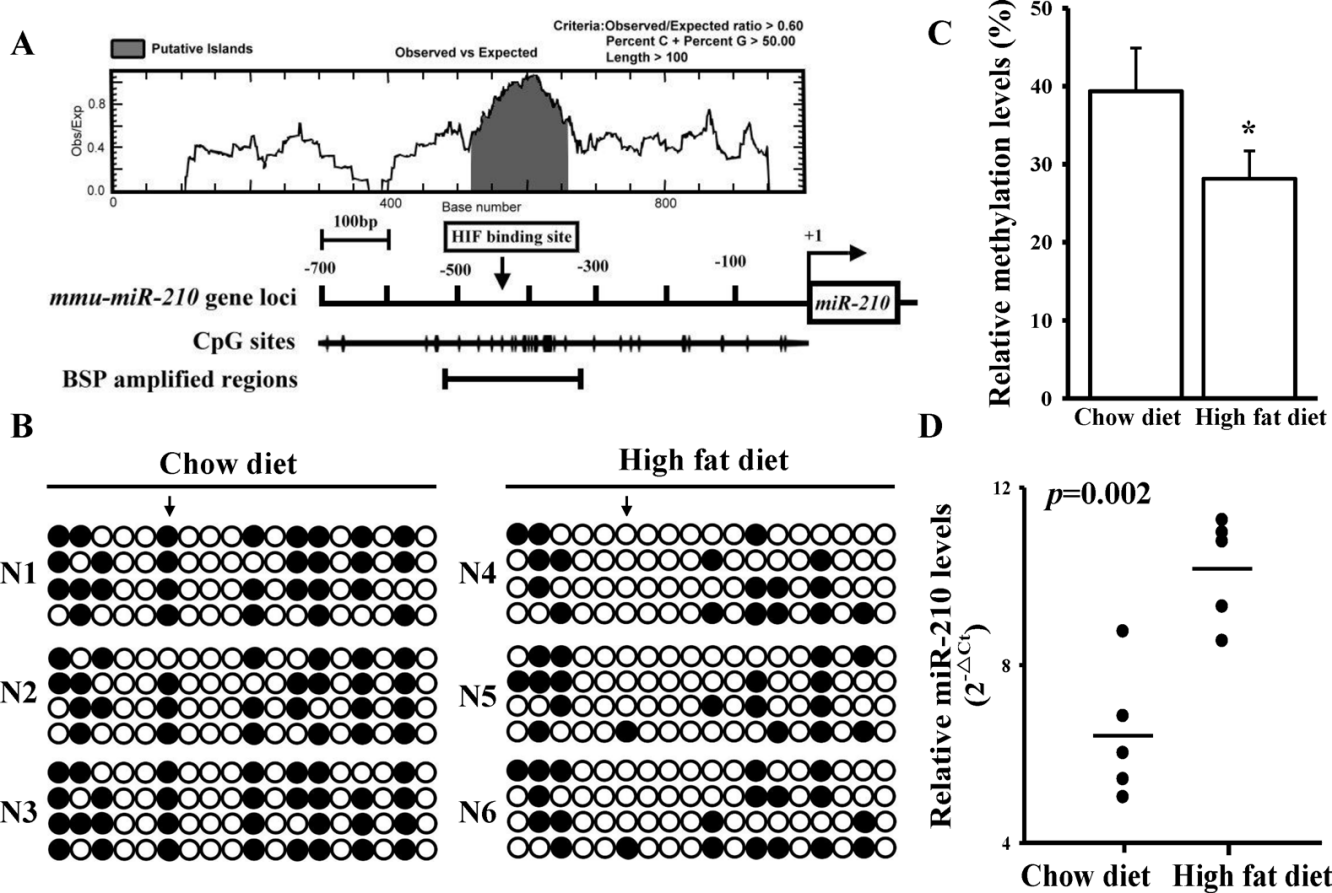
A high-fat diet reduces DNA methylation levels in the miR-210 promoter *in vivo* **A.** CpG island (gray color) and HIF-1α-binding site predictions in the mouse miR-210 gene promoter. Suitable primers were designed to amplify the CpG region containing the HIF-1α-binding site. **B.** The high-fat diet effects on DNA methylation in the mouse miR-210 gene promoter. The arrow indicates the HIF-1α-binding site. The N1 ∼ 3 and N4 ∼ 6 respectively means the serial number of control and high-fat diet-fed mouse. Each row and circle respectively means one single sequencing reaction and one CpG site. The empty and solid circles respectively mean the un-methyl and methyl CpG site. **C.** Quantitative results for. After being fed a high-fat diet, genomic DNA was extracted from the aorta of an APOE−/− mouse. The methylation status was measured by the BSP assay. **D.** The high-fat diet effects on miR-210 levels. Total RNA was extracted from aortas of mice fed a chow or high-fat diet, and miR-210 levels were measured by a qPCR. Data are means ± SD of three experiments. *P < 0.05.

We further tested for oxLDL's effect on the miR-210 promoter in mice. The serum lipid levels and aorta morphology were measured in mice fed with low or high fat diet (Suppl. Figure 1E–1G). First, a CpG island was predicted to occur in the murine miR-210 promoter region between −300 and −500 bp (Figure 3A). Furthermore, an HIF-1α-binding site was also located inside this CpG island. The BSP assay of this 200-bp region of murine genomic DNA extracted from the aorta showed that a high-fat diet decreased methylation levels from 40% to 28% (Figure 3B, 3C). Similarly, methylation levels at the HIF-1α-binding site were significantly lower in DNA extracted from the aorta of mice fed a high-fat diet. Consistent with findings from the cellular study, miR-210 expression levels were significantly higher in mice consuming a high-fat diet compared with those fed a chow diet (Figure 3D). Both *in vitro* and *in vivo* results demonstrated that oxLDL decreased the DNA methylation level leading to an increase in miR-210 expression.

### HIF-1α regulates miR-210 expression

**Figure 4:**
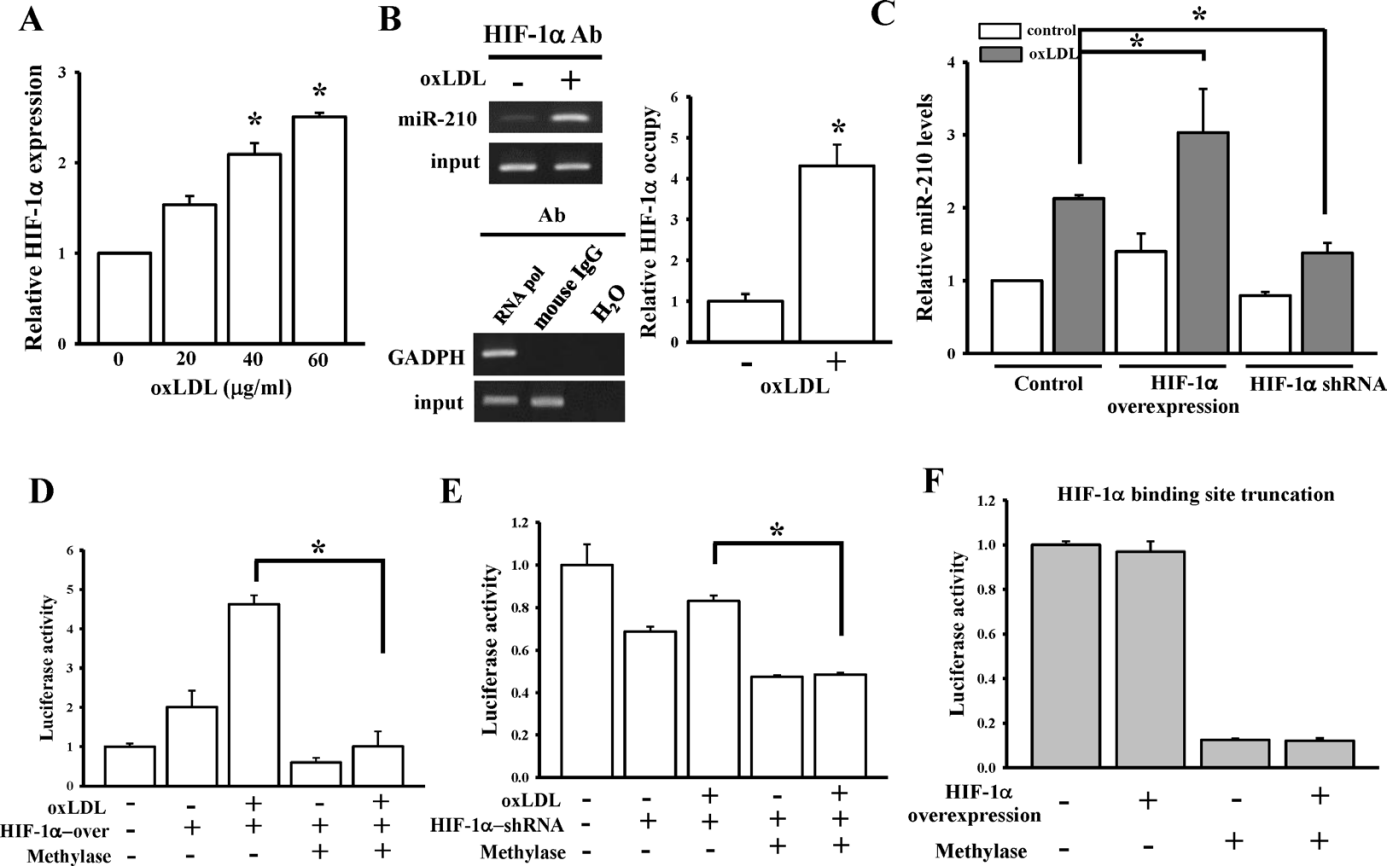
DNA methylation influences regulation of HIF-1α on miR-210 expression **A.** oxLDL effects on HIF-1α expression levels. HIF-1α expression levels were measured by a qPCR after treatment with different doses of oxLDL for 48 h. **B.** oxLDL effects on HIF-1α binding to the miR-210 promoter. After HASMCs were treated with 40 μg/ml oxLDL for 48 h, the HIF-1α-binding ability was measured by a ChIP assay. The right panel shows quantitative results from the ChIP assay. **C.** Overexpression or knockdown effects of HIF-1α on miR-210 levels. After transfection of full-length HIF-1α cDNA or HIF-1α shRNA into HASMCs and treatment with 40 μg/ml oxLDL for 48 h, the relative miR-210 expression was measured by a qPCR. The three white bars did not significantly differ. **D and E.** The effects of DNA methylation on HIF-1α-binding ability to the miR-210 promoter by *in vitro* methylation assay. HASMCs were co-transfected with a methylase-treated miR-210 promoter vector and full-length HIF-1α cDNA (or HIF-1α shRNA). After treating cells with 40 μg/ml oxLDL for 48 h, luciferase activity was measured in triplicate experiments. **F.** DNA methylation effects on miR-210 promoter activity with HIF-1α-binding site truncation. HASMCs were co-transfected with the methylase-treated miR-210 promoter vector in which the HIF-1α-binding site was truncated and full-length HIF-1α cDNA. After 48 h of incubation, luciferase activity was measured in triplicate experiments. Data are means ± SD of three experiments. *P < 0.05.

Previous studies showed that oxLDL induced HIF-1α accumulation in macrophages under normoxia [31]. We also found that oxLDL caused a dose-dependent increase in HIF-1α expression in HASMCs (Figure 4A). A Chromatin immunoprecipitation (ChIP) assay was conducted to investigate whether HIF-1α is involved in oxLDL-induced miR-210 overexpression. Results showed that oxLDL increased the binding of HIF-1α to the miR-210 promoter by 4-fold (Figure 4B). Furthermore, overexpression or knockdown of HIF-1α significantly affected oxLDL-mediated miR-210 upregulation (Figure 4C, Suppl. Figure 1H, 1I). Taken together, oxLDL enhanced miR-210 expression by modulating HIF-1α in multiple ways.

We conducted an *in vitro* methylation promoter assay to determine whether methylation can suppress HIF-1α's effect on miR-210. As shown in Figure 4D and 4E, individual treatment with oxLDL or HIF-1α significantly induced miR-210 promoter activity. However, the promoting effect by HIF-1α or oxLDL was almost completely abolished when hypermethylation occurred in the promoter region. Furthermore, when the HIF-1α-binding site in the promoter vector was truncated (Figure 4F), DNA methylation induced by methylase still suppressed miR-210 promoter activity. These data suggested that the DNA methylation status in the promoter CpG islands is a major determinant of miR-210 expression.

### SPRED2 is a miR-210 target gene

**Figure 5:**
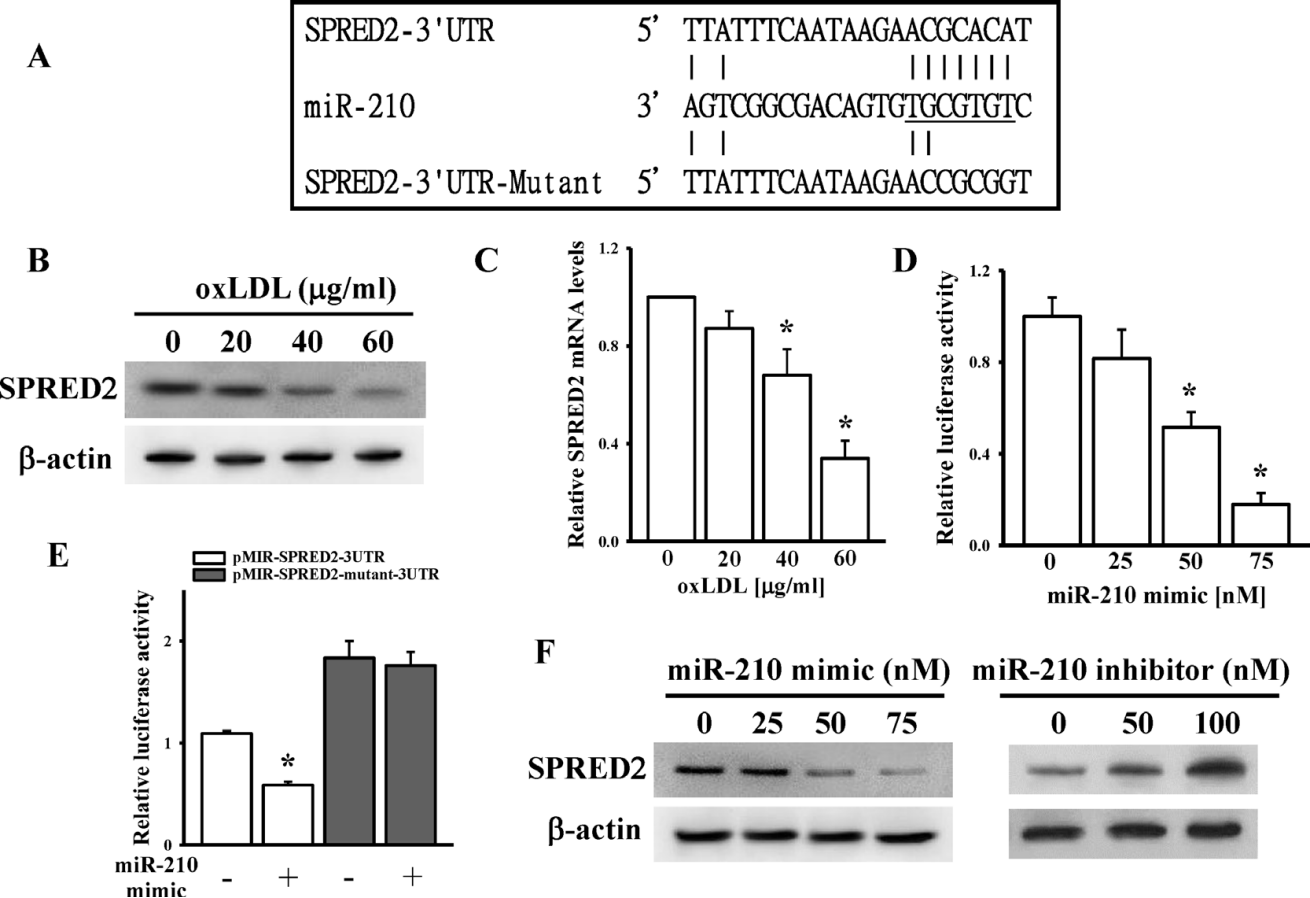
Identification of SPRED2 as a miR-210 direct target gene **A.** Schematic diagram shows the miR-210-binding site in the SPRED2 3′ UTR. **B and C.** oxLDL effects on SPRED2 protein and RNA levels. After treating HASMCs with different doses of oxLDL for 48 h, SPRED2 protein and RNA levels were measured by immunoblotting and a qPCR, respectively. **D and E.** The effects of miR-210 on SPRED2 3′-UTR luciferase activity. After cells had been co-transfected with the miR-210 mimic and pMIR-SPRED2–3UTR or mutant plasmids for 24 h, luciferase activity was measured in triplicate experiments. **F.** The miR-210 mimic and inhibitor effects on SPRED2 levels. After cells had been transfected with different doses of the miR-210 mimic and inhibitor for 48 h, SPRED2 protein levels were measured by immunoblotting assay. Data are means ± SD of three experiments. *P < 0.05.

The SPRED2 protein was identified as a key negative regulator of MAPK (mitogen-activated protein kinase) signaling in mammalian cells, which results in inhibition of cell migration [32]. A silicon analysis predicted that miR-210 could bind to the SPRED2 3′ UTR (Figure 5A). We first demonstrated that oxLDL reduced both mRNA and protein levels of SPRED2 in dose-dependent manners in HASMCs and HUVECs (Figure 5B, 5C, Suppl. Figure 2A, B). While SPRED2 expression was suppressed by oxLDL, other SPRED members (SPRED1 and −3) showed no compensatory upregulation (Suppl. Figure 2C, D). To confirm that SPRED2 is a miR-210 target gene, the full length (2648 bp) of the SPRED2 3′UTR was cloned into a pMIR-reporter plasmid. Different concentrations of the miR-210 mimic caused significantly decreases in luciferase activity (Figure 5D). When five nucleotides of the SPRED2 3′UTR corresponding to the miR-210 seed region were mutated by site-directed mutagenesis, miR-210 could no longer knock down luciferase activity (Figure 5E). Transfection of the miR-210 mimic and inhibitor into HASMCs significantly and dose-dependently influenced SPRED2 protein expression (Figure 5F).

### miR-210 affects phenotypes of vascular cells

To test for the effects of miR-210 on cell proliferation and migration, 50 nM of the miR-210 mimic, miR-210 inhibitor (i.e., antagomiR-210), and scrambled negative control were respectively transfected into HASMCs overnight. After 48 h of treatment with oxLDL (40 μg/ml), HASMC viability and migration were respectively measured by WST-1 and wound healing assays (Suppl. Figure 3A ∼ 3E). Overexpression of miR-210 enhanced oxLDL-mediated HASMC migration, and vice versa. However, miR-210 did not show a significant effect on HASMC proliferation (data not shown). Using the same study protocol, miR-210 was shown to increase HUVEC migration (Suppl. Figure 3F ∼ 3H). All results showed that miR-210 increased oxLDL-mediated HASMC and HUVEC migration.

### The role of SPRED2 in oxLDL-mediated cell migration

**Figure 6:**
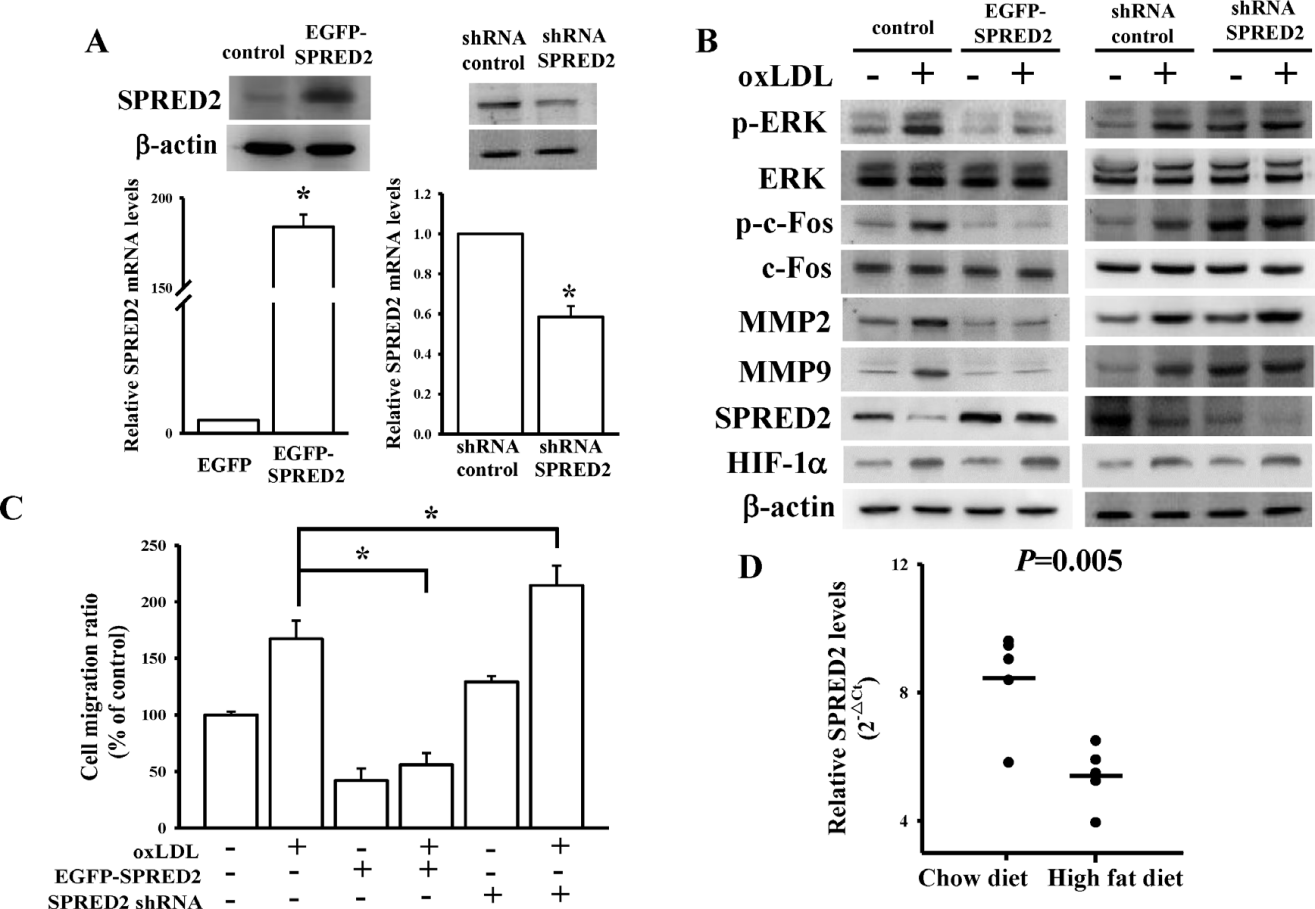
Identification of SPRED2 effects on oxLDL-induced HASMC migration **A.** Expression levels of SPRED2 after overexpression or knockdown of SPRED2 in HASMCs. The upper and lower panels respectively show protein and RNA levels of SPRED2. **B.** SPRED2 effects on oxLDL-mediated ERK/c-Fos/MMP-2/MMP-9 signaling pathway. **C.** Overexpression or knockdown effects of SPRED2 on oxLDL-mediated HASMC migration. After transfecting 1 μg of full-length SPRED2 cDNA or SPRED2-shRNA for 48 h, a cell migration experiment was conducted by a transwell migration assay. **D.** The high-fat diet effects on SPRED2 expression *in vivo*. Total RNA was extracted from the aorta of mice fed a regular chow or high-fat diet, and SPRED2 mRNA was measured by a quantitative real-time PCR. Data are means ± SD of three experiments. *P < 0.05.

Gain- and loss-of-function experiments were conducted using transient transfection of SPRED2 full-length cDNA without UTRs or SPRED2 shRNA to test the effect of SPRED2 on HASMCs (Figure 6A). There was no difference in phosphorylated extracellular signal-regulated kinase (p-ERK) levels between control (no plasmid) and EGFP (empty plasmid) group (data not shown). We found that SPRED2 could repress oxLDL-induced ERK phosphorylation, decrease the accompanying c-Fos phosphorylation, and downregulate matrix metalloproteinase (MMP)-2/MMP-9 expressions (Figure 6B). U0126, an ERK inhibitor, significantly attenuated the effects of SPRED2 knockdown on oxLDL-mediated ERK pathway (Suppl. Figure 3I). miR-210 inhibitor could also reduce the oxLDL effects on ERK/Fos/MMP pathway (Suppl. Figure 3J). Using a transwell migration assay, SPRED2 significantly suppressed oxLDL-mediated HASMC migration (Figure 6C, Suppl. Figure 3K). SPRED2 expression significantly decreased in mice fed a high-fat diet compared to those fed a chow diet (Figure 6D). The above data indicate that SPRED2 plays a critical role in oxLDL-mediated HASMC migration.

### DNA methylation of the miR-210 promoter and CVDs

**Figure 7:**
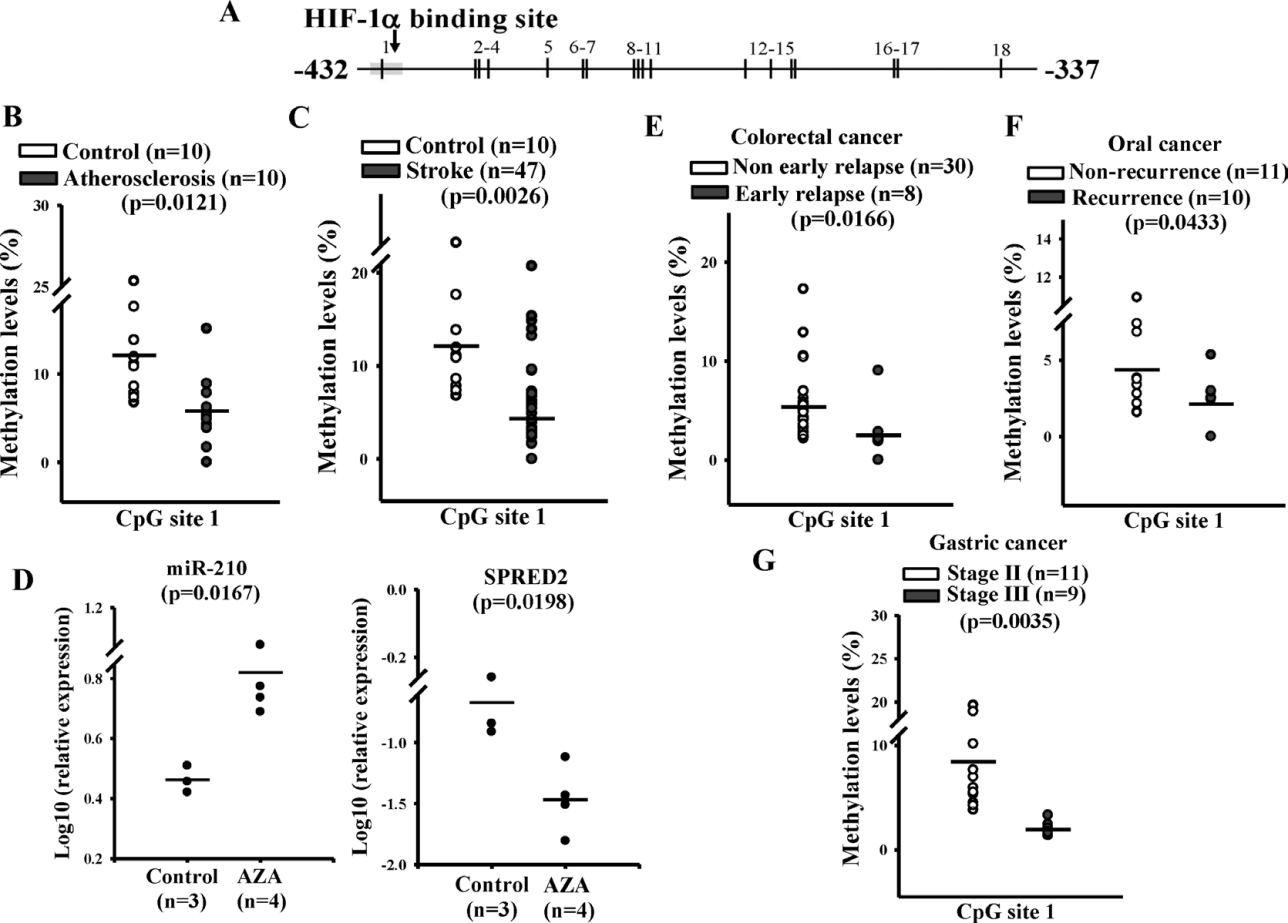
DNA methylation changes of the miR-210 promoter in patients with atherosclerosis, stroke, and gastrointestinal cancer **A.** Schematic diagram shows 18 CpG sites located between −432 and −337 bp in the miR-210 promoter. Methylation levels of these 18 CpG sites were individually measured by pyrosequencing. The fully Methylation results of these 18 CpG sites were showed in supplementary data. **B and C.** Methylation levels in the CpG site 1 of miR-210 promoter in atherosclerosis and stroke patients than controls. Genomic DNA was collected from the whole blood of control subjects (*n* = 10), and atherosclerosis (*n* = 10) and stroke (*n* = 47) patients. After bisulfite treatment and pyrosequencing, methylation levels were determined by PyroMark Q24 (Qiagen). **D.** Relative miR-210 and SPRED2 levels in AZA-treated xenograft animals. Mice with HT-29 xenografts were subcutaneously injected with PBS (*n* = 3) or 5 mg/kg AZA (*n* = 4) every 3 days. After treatment for 3 weeks, total RNA was extracted from the tumor. miR-210 and SPRED2 mRNA levels were determined by a quantitative real-time PCR. Data are means ± SD of three experiments. *P < 0.05. **E to G.** Methylation level changes in the CpG site 1 of miR-210 promoter in different cancer samples. Genomic DNA was collected from patients’ cancer tissues. After bisulfite treatment and pyrosequencing, methylation levels were determined by PyroMark Q24 (Qiagen). **E.** Non-early relapse (*n* = 30) vs. early relapse (*n* = 8) in colorectal cancer patients, **F.** non-recurrence (*n* = 11) vs. recurrence (*n* = 10) in oral cancer patients, and **G.** stage II (*n* = 11) vs. stage III (*n* = 9) gastric cancer. Any outlier > mean ± 3 SD needs to be removed.

To test whether aberrant DNA methylation of the miR-210 gene is implicated in CVDs, methylation levels were compared among healthy controls (*n* = 10), and atherosclerosis (*n* = 10) and stroke patients (*n* = 47). Genomic DNA was extracted from participants’ leukocytes, and bisulfite pyrosequencing was conducted to determine methylation levels. Since the R2 region contains an HIF-1α-binding site and also had a significant change in methylation levels (Figure 2B), we focused on 18 CpG sites of R2 located between −337 to −432 bp of the miR-210 promoter, especially the CpG site 1 (Figure 7A). The first CpG site is located at the HIF-1α-binding site. Average methylation levels from the 18 CpG sites were lower in both atherosclerosis (3.6%) and stroke patients (4.0%) than in the controls (6.3%) (Suppl. Figure 4A, 4B, Suppl. Tables 1, 2). The difference in methylation levels at the CpG site 1 was even more prominent between patients and controls: 5.8% in atherosclerosis patients, 5.8% in stroke patients, and 12.1% in the controls (Figure 7B, 7C).

### DNA methylation of the miR-210 promoter and cancers

Several studies reported that upregulation of miR-210 can enhance tumor cell proliferation and migration [25, 33]. HIF-1α also plays a critical role in tumorigenesis [34]. HT-29 cell is a cancer cell line commonly used in xenograft mice. oxLDL can also increase the expression of miR-210 (Suppl. Figure 5). Therefore, HT-29 was chosen for the xenograft model. To test the methylation effects of the miR-210 promoter on tumor formation *in vivo*, 5-aza-2′ deoxycytidine (AZA) was used to treat HT-29 xenograft mice. As shown in Figure 7D, AZA significantly induced miR-210 expression but reduced SPRED2 expression in HT-29 xenograft tumors. However, AZA significantly reduced rather than enhanced HIF-α expression (Suppl. Figure 6A and 6B). Given that AZA is used as an anticancer drug, not surprisingly, AZA treatment also caused a reduction in the tumor volume and mouse weight (Suppl. Figure 6C and 6D). We further tested whether methylation levels in the miR-210 promoter were associated with the aggressiveness of cancers. Three types of cancers were used in the present study because of their availability and relationship with dyslipidemia: relapse of colorectal cancer, relapse of oral cancer, and different stages of gastric cancer. Genomic DNA was obtained from frozen tumor tissues, and methylation levels between positions of −337 and −432 bp were measured by bisulfite pyrosequencing. The sample size of each type of cancer was described in “Methods”. Not all the blood samples were available for methylation measure from these cancer patients. In addition, the methylation data from the peripheral blood may not reflect what happened in the local tissues. We also acknowledged sample size from each cancer is relatively small and thus the results may need to be confirmed by other studies.

Methylation levels at the CpG site of the HIF-1α-binding site (CpG site 1) were significantly lower in patients with any type of a more-aggressive cancer, while methylation patterns at other CpG sites were inconsistent (Figure 7E–7G, Suppl. Figure 4C#x2013;4E, Suppl. Tables 3–5). Expression levels of miR-210 and SPRED2 also significantly differed between patients with more- and less-aggressive cancers, while HIF-1α expression levels did not differ (Suppl. Figure 7A–7C). The negative correlation was also observed between miR-210 and SPRED2 levels (Suppl. Figure 7D). The data suggest that high expression of miR-210 was associated with less-favorable prognoses, and high levels of SPRED2 indicated a good prognosis, while HIF-1α had no predictive value.

## DISCUSSION

**Figure 8:**
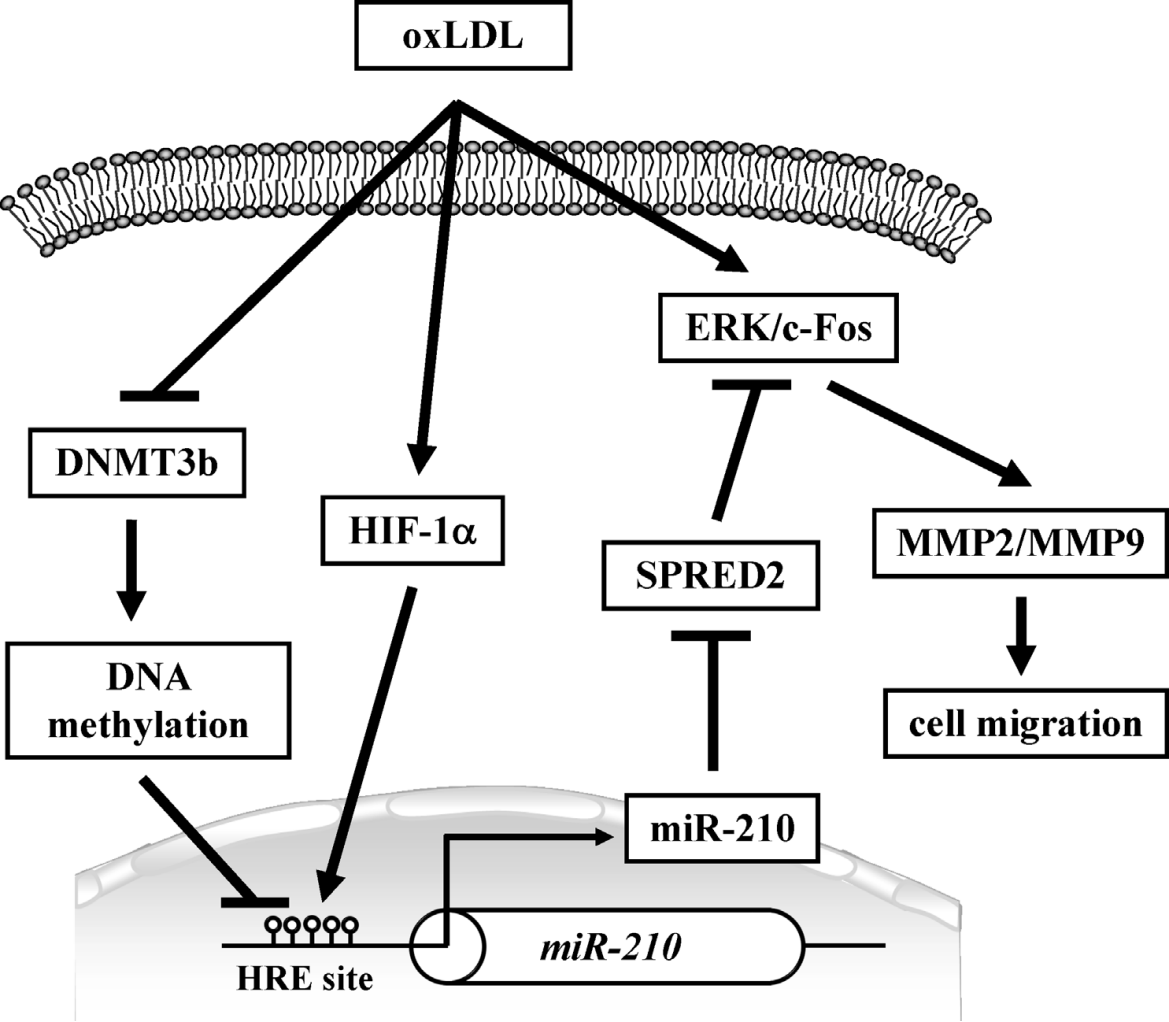
Schematic diagram shows that oxLDL decreases DNA methylation levels in the miR-210 gene promoter resulting in an increase of HIF-1α binding and miR-210 upregulation miR-210 directly inhibits SPRED2 expression, which promotes ERK/c-Fos signaling leading to cell migration.

The present study demonstrated that oxLDL can provoke both atherosclerosis and cancer progression via epigenomic regulation of miR-210 expression. There are three interlacing mechanisms (Figure 8) accounting for oxLDL-induced miR-210 overexpression: (1) oxLDL can decrease DNMT3b leading to DNA hypomethylation of the miR-210 promoter; (2) oxLDL can modulate HIF-1α levels that further increase miR-210 promoter activity; and (3) more interestingly, hypomethylation of the miR-210 promoter can facilitate HIF-1α binding to this region. We further demonstrated that oxLDL-induced miR-210 upregulation caused inhibition of SPRED2 transcription leading to activation of ERK/c-Fos/MMP-2/MMP-9 signaling. Animal studies using a xenograft mice model and ApoE KO mice fed a high-fat diet further supported the link among oxLDL, miR-210, and SPRED2 in relation to tumorigenesis and atherosclerosis. Our human studies using carotid atherosclerosis, stroke, and cancer patients showed that DNA hypomethylation of the miR-210 promoter was a common phenomenon in these diseases. Our results suggest that the miR-210 expression level has significant clinical relevance. In addition, our data provide an addition line of evidence to link cardiovascular and cancer risks.

Although the relationship between oxLDL and atherogenesis has been validated, oxLDL's effect on tumorigenesis was undisclosed until only recently [1, 9]. Previous studies reported that oxLDL binds to its receptor, LOX-1, to activate multiple downstream pathways to increase oxidative stress, inflammation, angiogenesis, and tumor dissemination [1, 2]. The present study further demonstrated a novel mechanism for oxLDL in the context of atherogenesis and tumorigenesis. Furthermore, several miRNAs have been identified in relation to cardiovascular diseases, among which some miRNAs are directly regulated by oxLDL. Given that oxLDL is a risk factor for both cardiovascular diseases and cancers, the oxLDL-regulated miRNAs can be involved in both disease categories. For examples, the let-7 [19], miR-1 [35] and miR-26 [36] are considered tumor suppressor miRNAs in gastrointestinal cancers, and their levels can be reduced by oxLDL. However, their clinical significance needs to be further explored and confirmed. Similar to our findings, upregulation of miR-210 was reported in colorectal cancer, and in early and advanced stages of gastric cancers and lung cancers [37–39]. A meta-analysis also showed that high miR-210 levels predicted a poor survival rate in breast cancer [40]. Elevated miR-210 expression was documented in atherosclerotic plaques of human arteries and the brain cortex of a stroke mouse model [23, 41]. Furthermore, our data provide more information to explain how miR-210 expression is regulated and how miR-210 affects SPRED2 to influence cancer prognoses and vascular diseases.

We showed that oxLDL inhibited DNMT3b leading to hypomethylation at multiple CpG sites of the miR-210 promoter. A recent study showed that hypoxia caused a decrease in DNMT3b levels and hypomethylation of the miR-210 promoter in neural progenitor cells. Previous studies found that HIF-1α can bind to the HRE of the miR-210 gene to activate miR-210 transcription in a variety of cancer types [25]. The discovery of the influence of the DNA methylation status on HIF-1α binding to its HRE is novel and further implies the complexity of genetic and epigenetic regulatory mechanisms. However, promoter activity for miR-210 expression has both HIF-1α-dependent and -independent mechanisms as shown in Figure 4D–4F.

The interaction between epigenetic modification and transcription factors could be a general and important regulatory mechanism in gene expression. Binding sites of transcription factors were reported in CpG islands [42]. For example, the methylation status of several promoter regions can alter the bindings of nuclear factor (NF)-κB and cAMP-responsive element binding (CREB)-1, which consequently affects gene transcription [43, 44]. In line with our findings, Wenger et al. found that hypermethylation of the HIF-1α-binding site in the erythropoietin gene promoter abolishes HIF-1α binding and gene activation.

Several miR-210 target genes have been reported to exert inhibitory effects on cell migration. These genes include Ephrin-A3 [45], Kruppel-like factor (KLF) 6 [46], Vacuole Membrane Protein (VMP) 1 [47], and PTPN2 [48]. However, the present study is not meant to comprehensively investigate all the miR-210 target genes. Instead, we tried to discover more unknown mechanisms to explain the phenotypes of interest. In our study, we identified SPRED2 as a novel target gene of miR-210. A number of studies have reported that overexpression of SPRED2 inhibits cell proliferation and migration through inactivating the Ras/Raf1/MAPK cascade [49, 50]. Downregulation of SPRED2 was reported in prostate cancer and hepatocellular carcinoma [51, 52]. Our data suggested that oxLDL-induced miR-210 can enhance SPRED2/ERK/c-Fos/MMP signaling, which explains oxLDL/miR-210′s effect on the three types of cancers tested in the present study.

In summary, the present study revealed an integrated network of an environmental factor (oxLDL), DNA methylation, a transcription factor, and microRNA in relation to vascular diseases and cancers (Figure 8). oxLDL decreases DMNT3b which causes hypomethylation of the miR-210 promoter, which in turn enhances HIF-1α binding, while oxLDL also upregulates HIF-α expression. Therefore, oxLDL can significantly increase miR-210 expression, which causes SPRED2 downregulation leading to a high risk of both cancer and vascular diseases. It has been reported that a combination of oxLDL and hypoxia is acting in an additive manner on HIF-1α protein content. It needs to notice that we performed all experiments under normoxia and it is unclear whether oxLDL has similar effect on miR-210 under hypoxia. We previously reported that miR-29b could inhibit DNMT3b expression [20], and the current study shows DNMT3b affects miR-210. The complex regulatory network among miR-29b, miR-210 and DNMT3b needs further investigation. The novel findings provide additional evidence to link atherosclerotic vascular diseases and cancers.

## MATERIALS AND METHODS

Expanded Materials and Methods can be found in the Online Data Supplement.

### Cell culture, treatments, and transfections

Primary HASMCs were grown in medium 231 supplemented with SMGS at 37°C in a humidified atmosphere of 95% air/5% CO_2_. Cells between passages 6 ∼ 10 were used in all experiments. For AZA treatment, cells were treated with 2 μM or a different dose for 48 h. The methods for oxLDL treatment and transfections were detailed as previously [19].

### *In vitro* methylation of miR-210 promoter

Plasmids containing the miR210 promoter were methylated using SssI methylases (Zymo Research; Irvine, CA, USA) according to the manufacturer's instructions. In brief, 1 μg of plasmid DNA was incubated with Sss I methylases in the presence of 600 μM S-adenosylmethionine for 2 h at 30°C. The control group using the same plasmid was processed in the same way except for methylase treatment. For the reporter assay, HASMCs were transiently transfected with a reporter plasmid (methylated or unmethylated) and an HIF-1α expression plasmid or HIF-1α shRNA using Lipofectamine 2000 (Invitrogen; Waltham, MA, USA) in the presence of oxLDL. pEGFP plasmids were co-transfected and acted as the internal control. The reporter assay was performed at 24 h post-transfection using the Luciferase Assay System (Promega; Madison, WI, USA).

### ChIP assay

ChIP assays (EZ-ChIPTM, Millipore; Billerica, MA, USA) were carried out as described previously [19]. DNA recovered from samples containing the HIF-1α antibody was compared to the negative control (mouse immunoglobulin G (IgG)) and the positive control (an anti-RNA-Pol antibody) provided by the manufacturer. Finally, DNA was subjected to a PCR analysis after being recovered. The PCR primers are given in Suppl. Table 6.

### *In vivo* study for gene expression and methylation assay

APOE−/− mice were purchased from Jackson Laboratory (Bar Harbor, ME, USA). Mice were maintained in a temperature-controlled (25°C) facility with a strict 12-h light: dark cycle. All animals were allowed to adapt to the environment for at least 2 weeks prior to dietary treatment and were provided free access to food and water throughout the experiment. Weight gain was monitored every week, and food intake was determined two times per day during the period of each study. Male mice were fed either rodent chow (*n* = 10) or a high-fat diet (*n* = 10) that is known to elicit fatty streak lesions in the aortas of APOE−/− mice [60]. Mice were fed 7 g/day of the chow diet or high-fat diet for 6 weeks. After being sacrificed, total RNA and genomic DNA were extracted from the aorta. Gene expression levels were determined by a quantitative real-time PCR (qPCR). Genomic DNA was used for the methylation study.

### Bisulfite treatment, MSP and BSP

Bisulfite conversion of genomic DNA (200 ng) was performed with the EZ DNA Methylation Gold Kit according to the manufacturer's instructions (Zymo Research; Irvine, CA, USA). DNA was suspended in 10 μl of H_2_O and stored at −20°C. Primers for the MSP experiment in the miR-210 promoter are listed in Suppl. Table 6. PCRs (40 cycles) were performed using denaturation at 95°C for 30 s, annealing at 55°C for 30 s, and elongation at 72°C for 30 s. Products were separated by gel electrophoresis. For the BSP assay, the primers designed to specifically amplify miR-210 promoter regions are listed in Suppl. Table 6. PCR products were then cloned into the pcDNA3 vector. The 10 ∼ 15 clones from each sample were randomly selected for DNA sequencing. After sequencing, the four to six correct clones were used to measure methylation levels.

### Pyrosequencing of human samples

To study methylation changes in CVDs, genomic DNA was extracted from human blood samples of normal controls (*n* = 10), and atherosclerosis (*n* = 10) and stroke patients (*n* = 47). These samples were collected from the patients with age- and sex-matched in the same time period. For cancer research, genomic DNA was extracted from human tissue samples of colorectal cancer patients with non-early relapse (*n* = 30) and with early relapse (*n* = 8); oral cancer patients without (*n* = 11) and with recurrence in 5 years (*n* = 10); and gastric cancer patients at stage II (*n* = 11) and at stage III (*n* = 9). Early relapse of colorectal cancer was defined as local recurrence (tumor growth restricted to the anastomosis or the region of the primary operation) or distant metastasis (distant metastasis or diffuse peritoneal seeding) occurring within 1 year of radical resection [61].

DNA was extracted using a Qiagen DNeasy Blood and Tissue kit (Germantown, MD, USA) according to the manufacturer's instructions. Bisulfite conversion was conducted using an Qiagen EpiTect^®^ Bisulfite kit (Germantown, MD, USA) according to the manufacturer's instructions. The methylation status of the miR-210 promoter was assessed using a pyrosequencing-based methylation analysis. Primers for pyrosequencing listed in Suppl. Table 6 were designed using Qiagen Pyrosequencing Assay Design (Germantown, MD, USA). First, the PCR was carried out with Qiagen HotStarTaqPlus Master Mix (Germantown, MD, USA) to label bisulfite-converted DNA with biotinylated primers. The PCR conditions were 40 cycles of denaturation at 94°C for 30 s, annealing at 54°C for 30 s, and extension at 72°C for 30 s. After the PCR, the biotinylated strand was captured on streptavidin-coated beads (Amersham Bioscience, Uppsala, Sweden) and incubated with sequencing primers. Pyrosequencing was performed on a PSQ HS 24 pyrosequencing machine (Biotage, Uppsala, Sweden). The difference in methylation levels between patients with good and poor prognoses was analyzed by the PyroMark Q24 Software. Any outlier > mean ± 3 SD needs to be removed.

### *In vivo* xenograft study

Six-week-old nude mice (BALB/c nu/nu; National Laboratory Animal Center, Taipei, Taiwan) were housed in a sterile environment (in a specific pathogen-free room) with a light/dark cycle of 12/12 h. All animals were allowed to adapt to the environment for at least 2 weeks prior to dietary treatment and were provided free access to food and water throughout the experiment. HT29 cells in 0.1 ml Dulbecco's modified Eagle medium (DMEM) were subcutaneously (s.c.) injected into the right hind flank of the mice. Tumor sizes were measured daily with calipers and were calculated as 1/2 × length × width^2^ in mm^3^. When the tumors had grown to about 100 mm^3^, 1× PBS (control; *n* = 5) or 5 mg/kg AZA (*n* = 5) was injected into the tumor every 3 days for 3 weeks. Weight gain and tumor sizes were respectively monitored every day. At the end of the experiments, the mice were sacrificed, and tumor tissues were excised. Tissues were homogenized to extract the total RNA for further investigation.

### Statistical analysis

Student's *t*-test was used to compare all experimental results. A *p* value of < 0.05 was considered significant.

## SUPPLEMENTARY DATA


