# Supplementary material for: Oxidized low-density lipoprotein is a common risk factor for cardiovascular diseases and gastroenterological cancers via epigenomical regulation of microRNA-210
Source: Oncotarget. 2015 Jun 3;6(27):24105–18. doi: 10.18632/oncotarget.4152 (PMC4695173; doi:10.18632/oncotarget.4152)
Supplement: Supplementary file 1 [file oncotarget-06-24105-s001.pdf]

SUPPLEMENTARY DATA

Materials

Primary human aortic smooth muscle cells (HASMCs), medium 231, and smooth muscle cell growth supplement (SMGS) were purchased from Cascade Biologics (Portland, OR, USA). Other cell culture-related reagents were purchased from GIBCO-BRL (Grand Island, NY, USA). oxLDL was purchased from Biomedical Technologies (Ward Hill, MA, USA). An anti-matrix metalloproteinase (MMP)-2 antibody was purchased from Epitomics (Cambridge, UK). Anti-MMP-9, HIF-1α, and SPRED2 antibodies were purchased from GeneTex (Hsinchu City, Taiwan). Anti-phosphorylated ERK, ERK, phosphorylated c-Fos, and c-Fos antibodies were purchased from Cell Signaling Technology (Danvers, MA, USA). An anti-β-actin antibody and enhanced chemiluminescence (ECL) solution were purchased from Millipore (Billerica, MA, USA). Trizol® reagent, lipofectamine, secondary antibodies, SYBR® Green PCR Master Mix, MultiScribe(tm) Reverse Transcriptase Kit, PCR Master Mix, TaqMan® MiR-210 and U44 assays, the miR-210 mimic, and antagomiR were purchased from Thermo Fisher Scientific (Waltham, MA, USA). Primer sets were synthesized by Mission Biotech (Nankang, Taipei, Taiwan). An EZ DNA Methylation-Gold™ Kit was purchased from Zymo Research (Irvine, CA, USA). A genomic DNA isolation kit and EpiTect Bisulfite Kit for the pyrosequencing assay were purchased from Qiagen (Germantown, MD, USA). HIF-1α, SPRED2, DNMT3a, and DNMT3b short hairpin (sh)RNAs were purchased from the National RNAi Core Facility (Nankang, Taipei, Taiwan). Unless otherwise specified, all other reagents were of analytical grade.

Cell culture, treatments, and transfections

Primary HASMCs were grown in medium 231 supplemented with SMGS at 37°C in a humidified atmosphere of 95% air/5% CO_2. Cells between passages 6 ~ 10 were used in all experiments. For 5-aza-2' deoxycytidine (AZA, Sigma-Aldrich, St. Louis, MO, USA) treatment, cells were treated with 2 μM or a different dose for 48 h. The methods for oxLDL treatment and transfections were detailed as previously (*1*).

RNA isolation, quantitative real-time reverse transcription-polymerase chain reaction (PCR), and immunoblotting analysis

Total RNA extraction from cell culture or mouse aorta was carried out using Trizol® according to the manufacturer's instructions. Complementary (c)DNA was synthesized from 1 μg of total RNA using a random primer and the MultiScribe(tm) Reverse Transcriptase Kit. For miR-210 and U44 detection, cDNA was synthesized using the TaqMan® MicroRNA Assay. For the quantitative real-time PCR, specific primers for human HIF-1α, SPRED1, SPRED2, and SPRED3, and GAPDH were designed and are listed in Suppl. Table 6. Gene expression was measured with preoptimized conditions using an ABI 7900 real-time PCR machine (Applied Biosystems, Waltham, MA, USA). For detecting protein expression levels, total proteins were collected from cells and tissues. The methods for quantitative real-time PCR and immunoblotting assay were detailed as previously (*1*).

Transwell migration assays and Wound healing assay

To conduct the transwell migration assays, 5×10^4 cells were placed in the top chamber of a transwell migration chamber (8 μm; Millipore, Billerica, MA, USA). The detail methods of transwell migration assays and wound healing assay were carried out as previously described (*2*).

Construction of the SPRED2 3'UTR reporter plasmid and mutagenesis

A PCR was performed using the primers (shown in Suppl. Table 6) specifically for the SPRED2 3'UTR, of which the forward primer was SpeI-site-linked and the reverse primer was MluI-site-linked. HASMC genomic DNA was used as the template. PCR products were digested with SpeI/MluI and cloned downstream of the luciferase gene in the pMIR-REPORT luciferase vector (Ambion, Waltham, MA, USA). This vector was sequenced and named pMIR-SPRED2-3UTR. Site-directed mutagenesis of the miR-210 target-site in the SPRED2 3'UTR was carried out using a QuikChange® Site-Directed Mutagenesis Kit (Stratagene, Heidelberg, Germany) and named pMIR-SPRED2-mutant 3UTR, in which pMIR-SPRED2-3UTR was used as a template. The methods for reporter assay were detailed as previously (*1*).

Construction of the miR-210 promoter reporter plasmid

To construct the miR-210 promoter reporter plasmid, a 550-bp fragment of the miR-210 promoter containing the HIF-α-binding site was isolated by a PCR (the primers are listed in Suppl. Table 6). After digestion of the PCR product with XhoI and HindIII, the insert was cloned into the pGL3 reporter vector (Promega, Madison, WI, USA) to create the pGL3-miR-210 promoter vector.

An overlapping PCR was carried out to construct the miR-210 promoter reporter plasmid where the HIF-1α-binding site was truncated. In this procedure, the pGL3-miR-210 promoter vector was used as a template with suitable primers listed in Suppl. Table 6. All plasmid sequences were confirmed by DNA sequencing.

Statistical analysis

Student's *t*-test was used to compare all experimental results. A p value of < 0.05 was considered significant.

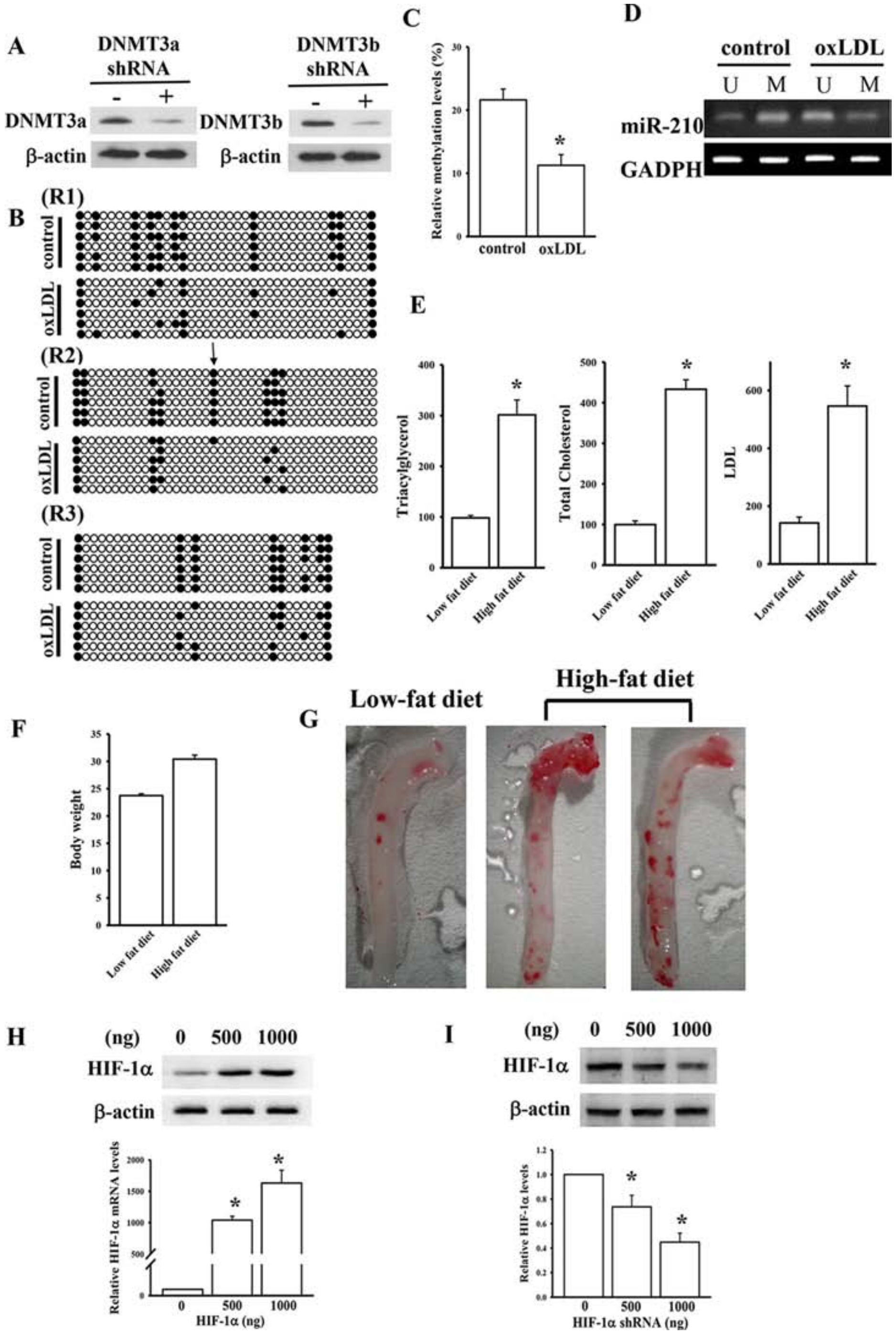

Supplementary Figure S1: oxLDL effects on DNA demethylation in miR-210 gene promoter of HUVEC cells. A. The knockdown efficiency of DNMT3a and 3b shRNA in HASMC cells. **B.** oxLDL effects on DNA demethylation in miR-210 gene promoter by BSP assay. The arrow means HIF-1α binding site. **C.** The quantitative results from figure (B). **D.** DNA methylation changes of miR-210 gene promoter by MSP assay. After HUVEC cells were treated with 40 µg/ml oxLDL for 48 h, the genomic DNA was extracted. Methylation status was determined by BSP and MSP assay, respectively. * means $p < 0.05$. **E.** The serum lipids levels, **F.** body weight changes, and **G.** arota morphology were measured in mice fed with low or high fat diet. **H** and **I.** The expression levels of HIF-1α after overexpression or knockdown of HIF-1α levels in HASMC. After transfecting with different dose of HIF-1α full length cDNA (H) or shRNA (I) for 48 h, the HIF-1α protein and RNA levels were measured by immunoblot and quantitative real-time PCR, respectively. The upper and lower figures respectively show the protein and RNA levels of HIF-1α expression. Data are means ± SD of three experiments. *$P < 0.05$.

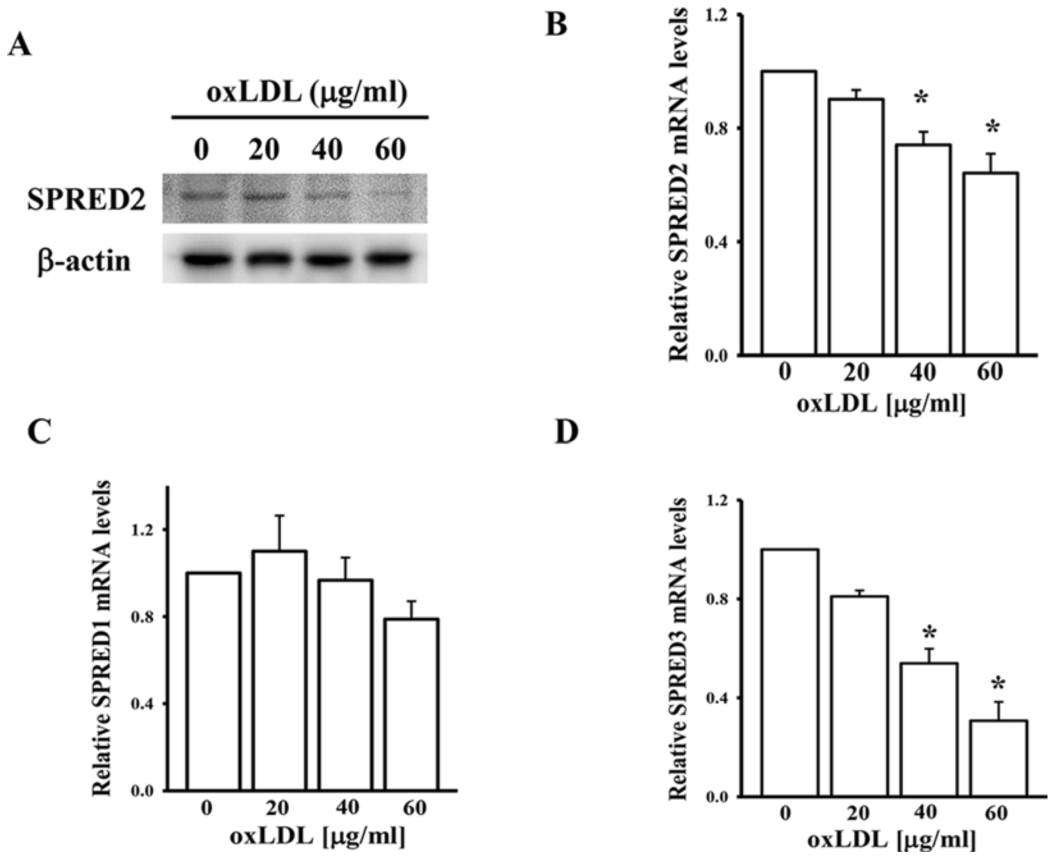

Supplementary Figure S2: oxLDL effects on SPRED2 expression levels in HUVEC cells. The effects of oxLDL on SPRED2 protein **A.** and RNA **B.** levels. After treating with different dose of oxLDL for 48 h, the SPRED2 protein and RNA levels were measured by immunoblot and quantitative real-time PCR, respectively. * means $p < 0.05$. **C** and **D.** oxLDL effects on SPRED isoforms levels. After treating with different dose of oxLDL for 48h, the SPRED1 (C) and SPRED3 (D) RNA levels were measured by quantitative real-time PCR. Data are means ± SD of three experiments. *$P < 0.05$.

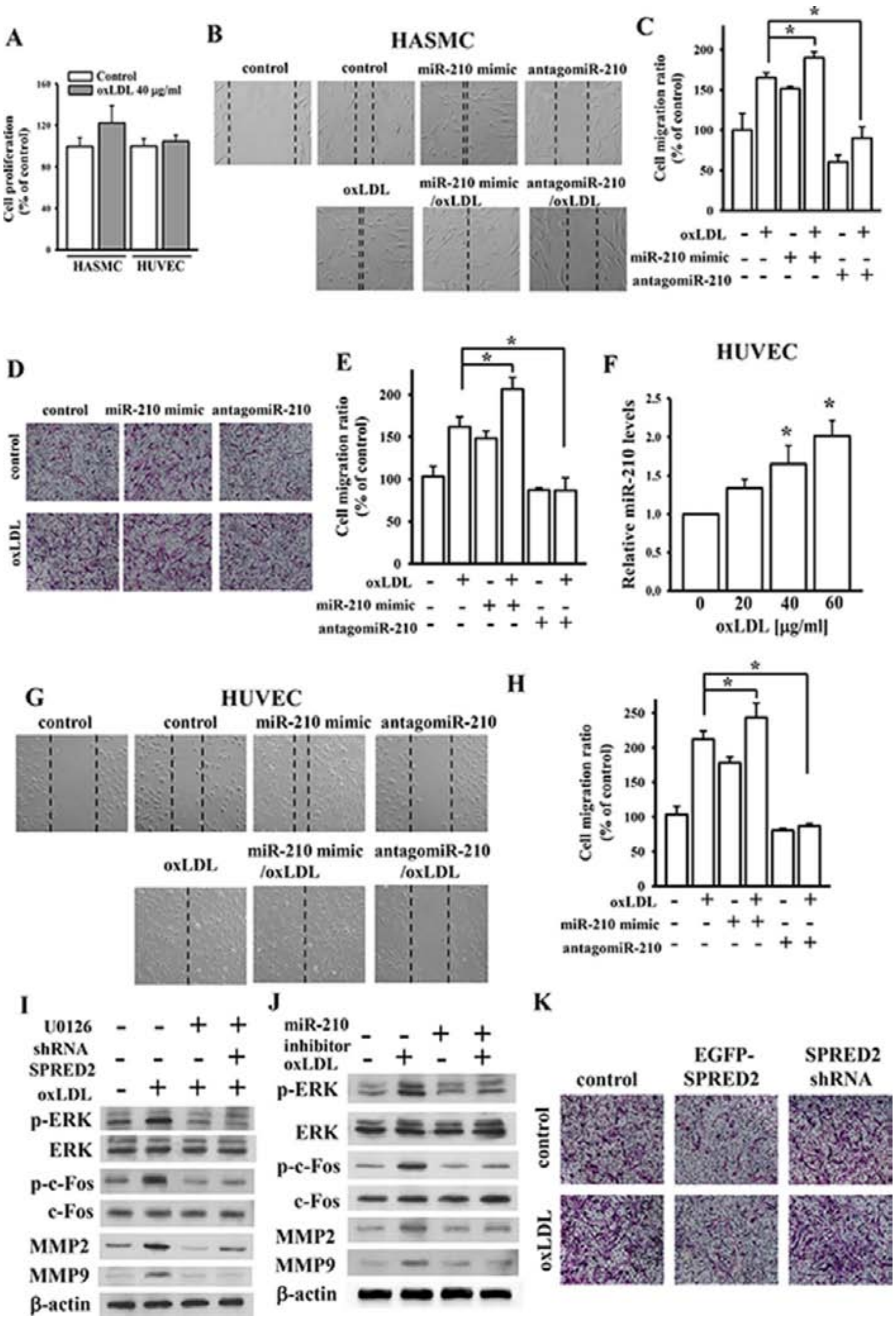

Supplementary Figure S3: miR-210 effects on oxLDL-induced cell migration. **A.** oxLDL effects on cell proliferation. **B.** The effects of miR-210 on oxLDL-mediated HASMC cell migration. After cells were transfected with 50nM miR-210 mimic or antagomiR-210 overnight, 40 µg/ml oxLDL was treated for 48 h. Cell migration changes were detected by wound healing assay. **C.** The quantitative results from wound healing assay in figure (B). **D.** oxLDL and miR-210 effects on HASMC cell migration by transwell migration assay. **E.** The quantitative results from transwell migration assay in figure (D). **F.** oxLDL effects on miR-210 expression in HUVEC cells. After treating with different dose of oxLDL for 48 h, the miR-210 expression levels were measured by quantitative real-time PCR. Data are means ± SD of three experiments. $^{*}P < 0.05$. **G.** The effects of miR-210 on oxLDL-mediated HUVEC cell migration. **H.** The quantitative results from wound healing assay in figure (G). After cells were transfected with 50 nM miR-210 mimic or antagomiR-210 overnight, 40 µg/ml oxLDL was treated for 48 h. Cell migration changes were detected by wound healing assay. **I.** Effects of U0126 and SPRED2 knockdown on oxLDL-mediated ERK pathway. **J.** Effects of miR-210 inhibitor on oxLDL-mediated ERK pathway. **K.** Overexpression or knockdown effects of SPRED2 levels on oxLDL-mediated HASMC cell migration. After transfecting with 1µg SPRED2 full length cDNA or shRNA for 48h, the cell migration changes were conducted and measured by transwell migration assay.

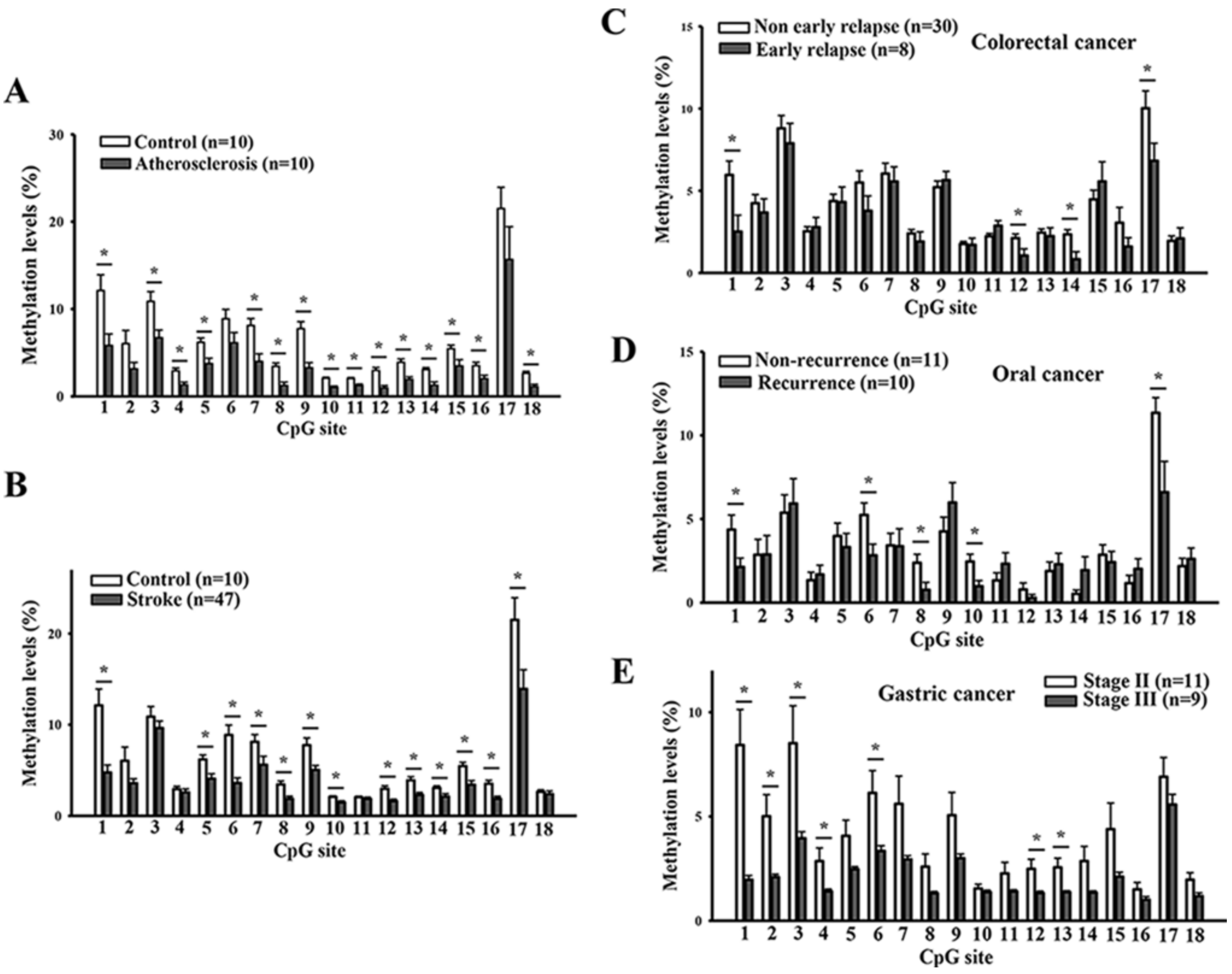

Supplementary Figure S4: DNA methylation changes in the miR-210 promoter in patients with atherosclerosis, stroke, and cancer. There are 18 CpG sites located between −432 and −337 bp in the miR-210 promoter. Methylation levels of these 18 CpG sites were individually measured by pyrosequencing. **A** and **B.** Methylation levels of miR-210 promoter in atherosclerosis and stroke patients than controls. Genomic DNA was collected from the whole blood of control subjects (n = 10), and atherosclerosis (n = 10) and stroke (n = 47) patients. After bisulfite treatment and pyrosequencing, methylation levels were determined by PyroMark Q24 (Qiagen). Data are means ± SD of three experiments. *$P < 0.05$. **C** to **E.** Methylation levels of the miR-210 gene promoter in different cancer samples. Genomic DNA was collected from patients' cancer tissues. After bisulfite treatment and pyrosequencing, methylation levels were determined by PyroMark Q24 (Qiagen). (C) Non-early relapse (n = 30) vs. early relapse (n = 8) in colorectal cancer patients, (D**)** non-recurrence (n = 11) vs. recurrence (n = 10) in oral cancer patients, and (E) stage II (n = 11) vs. stage III (n = 9) gastric cancer. Data are means ± SD of three experiments. *$P < 0.05$. Any outlier > mean ± 3 SD needs to be removed.

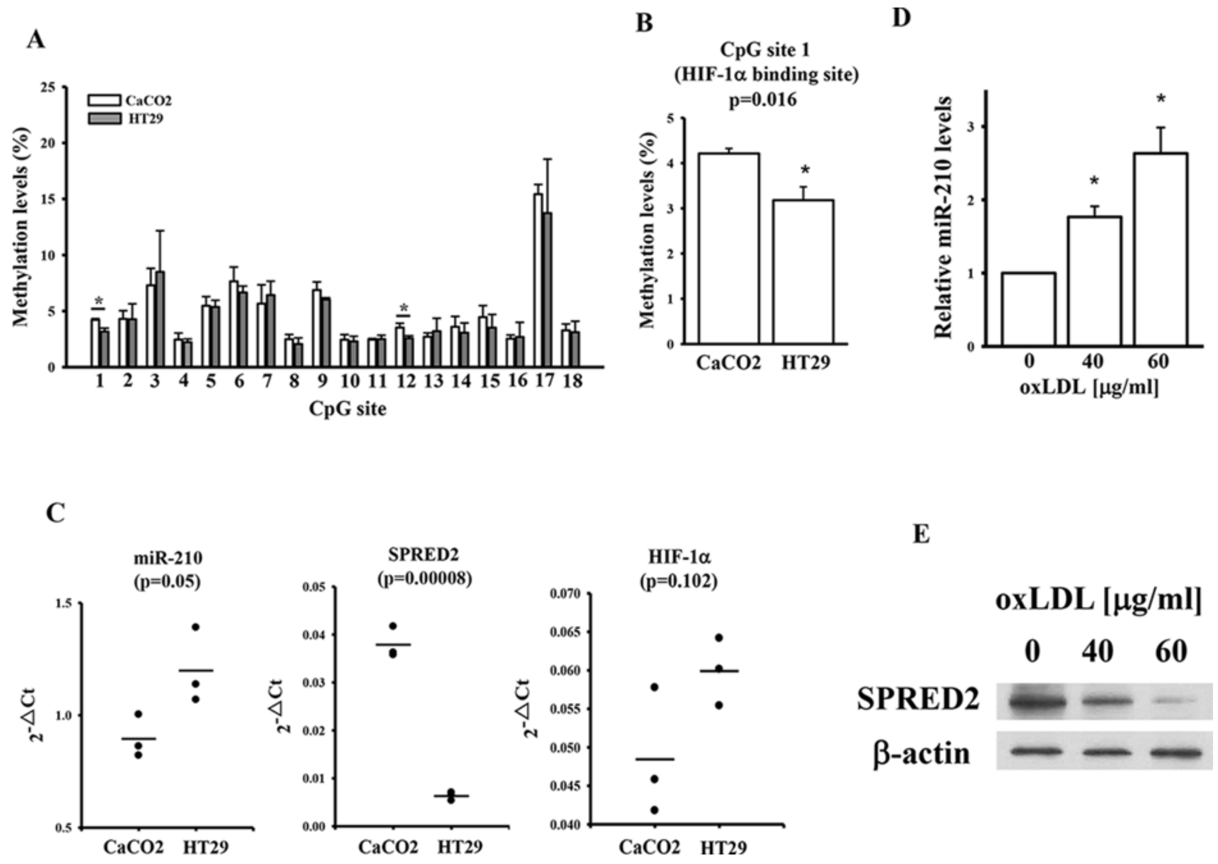

Supplementary Figure S5: Methylation effects on miR-210 levels in colon cancer cells. A. Methylation levels of 18 CpG sites in miR-210 promoter. **B.** Different methylation levels in the CpG site 1 of miR-210 promoter in caco2 and HT-29 cells. **C.** Relative miR-210, SPRED2, and HIF-1α expression levels in colon cancer cells. The effects of oxLDL treatment on miR-210 **D.** and SPRED2 **E.** expression levels in HT-29 cells. Data are means ± SD of three experiments. $^*P < 0.05$.

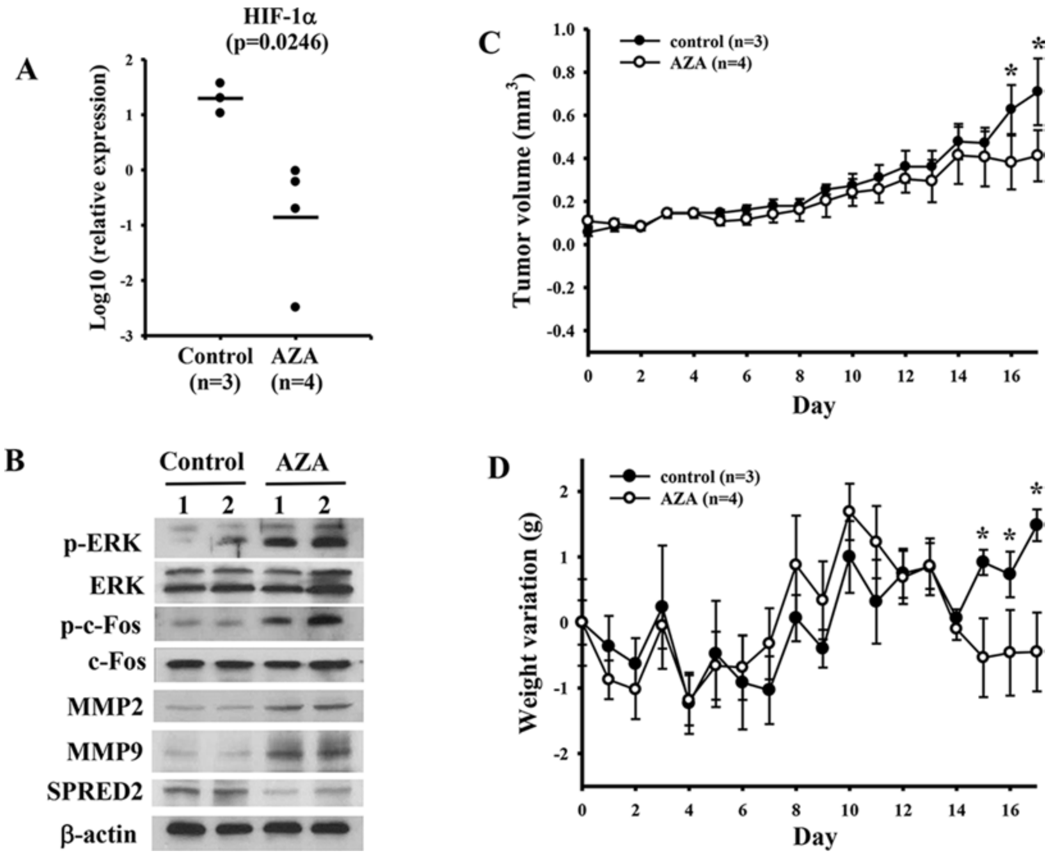

Supplementary Figure S6: The effects of AZA treatment on HIF-1αexpression levels, ERK-mediated pathway, tumor volume and weight variation in xenograft animal model. **A.** HIF-1αexpression levels. **B.** phosphorylation levels of ERK and c-Fos, MMP2, MMP9 and SPRED2 protein levels in xenograft tumors. **C.** tumor volume **D.** weight variation. Mice with HT-29 xenografts were injected every 3 days with PBS (n = 3) and 5 mg/kg AZA (n = 4). The tumor volume and mouse weight are respectively measured everyday. After treatment for 3 weeks, total RNAs were extracted from tumor tissue. The HIF-1αgene expression levels were determined by quantitative real-time PCR. Data are means ± SD of three experiments. *$P < 0.05$.

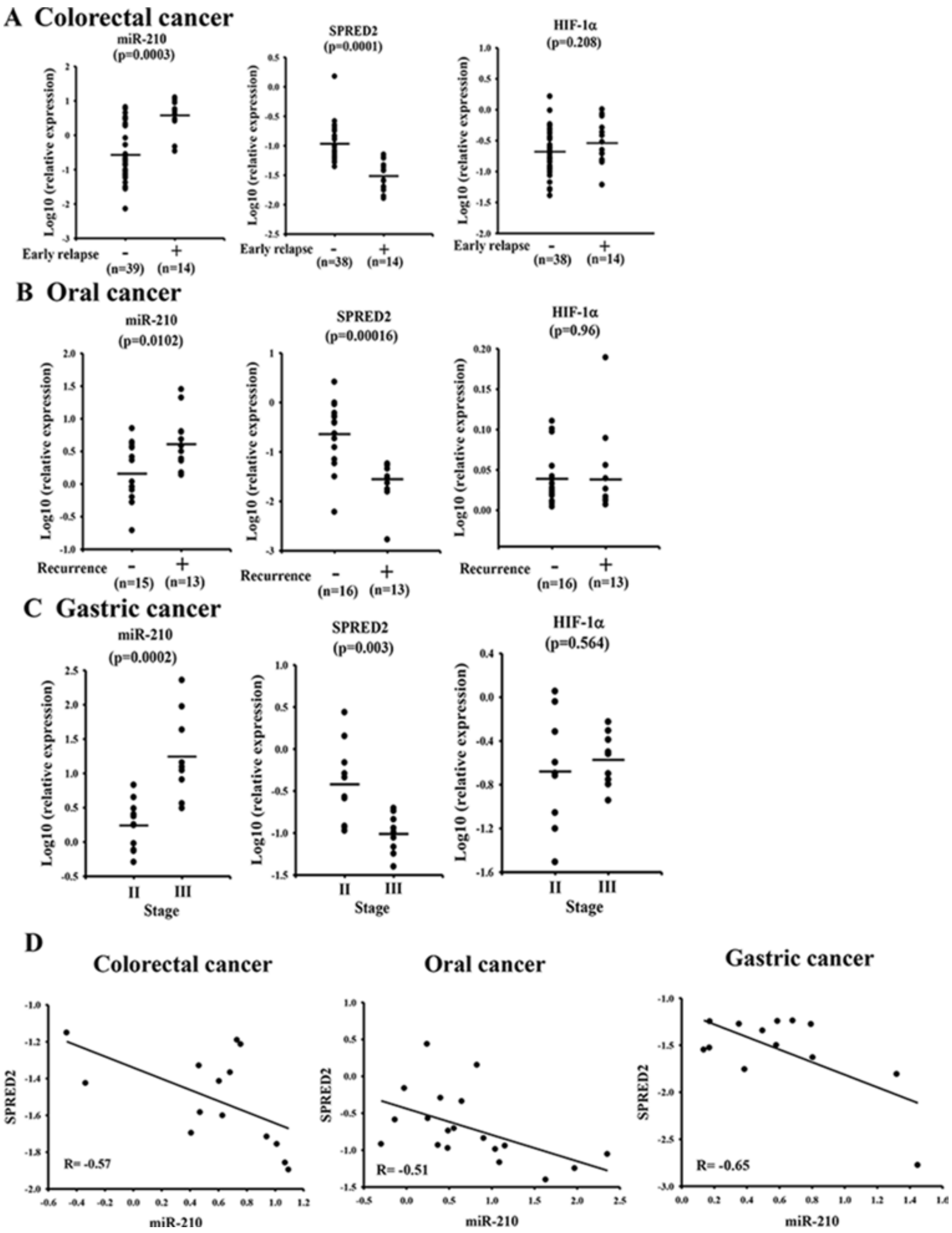

Supplementary Figure S7: The expression levels of miR-210, SPRED2, and HIF-1α in colorectal cancer, oral cancer and gastric cancer patients. Total RNA was extracted from tumor tissues. The relative expression levels of miR-210, SPRED2, and HIF-1α were respectively measured by quantitative real-time PCR. **A.** non early relapse (n = 38) V.S. early relapse (n = 14) in colorectal cancer patients. **B.** non-recurrence (n = 16) V.S. recurrence (n = 13) in oral cancer patients. **C.** stage II (n = 11) V.S. stage III (n = 9) in gastric cancer patients. **D.** The correlation between miR-210 and SPRED2 levels. Data are means ± SD of three experiments. *$P < 0.05$.

Supplementary Table S1. DNA methylation changes of miR-210 gene promoter from atherosclerosis patients comparing with control subjects

Atherosclerosis			
**CpG site**	**Control Subjects (*n* = 10)**	**Disease Subjects (*n* = 10)**	***P* Value**
1 (HIF binding site)	12.1 (5.7)	5.8 (4.2)	**0.012**
2	6.0 (4.8)	3.1 (2.3)	0.108
3	10.9 (3.5)	6.7 (2.8)	**0.009**
4	2.9 (1.0)	1.3 (0.9)	**0.001**
5	6.2 (1.6)	3.7 (2.0)	**0.007**
6	8.9 (3.4)	6.1 (3.7)	0.101
7	8.1 (2.5)	4.0 (2.8)	**0.003**
8	3.4 (1.2)	1.2 (1.2)	**0.001**
9	7.8 (2.5)	3.3 (1.9)	**0.001**
10	2.1 (0.3)	1.0 (0.5)	**2.698E-05**
11	2.0 (0.3)	1.2 (0.5)	**0.001**
12	3.0 (1.0)	1.0 (0.8)	**1.61E-04**
13	3.9 (1.2)	1.9 (0.9)	**0.001**
14	3.1 (0.7)	1.3 (1.1)	**0.001**
15	5.5 (1.3)	3.5 (2.3)	**0.030**
16	3.5 (1.2)	2.0 (1.2)	**0.012**
17	21.5 (7.7)	15.7 (11.9)	0.211
18	2.6 (0.7)	1.1 (0.9)	**0.001**
Average	6.3 (4.9)	3.6 (3.5)	0.063

Significant ($P < 0.05$) values are in bold.

Supplementary Table S2. DNA methylation changes of miR-210 gene promoter from Stroke patients comparing with control subjects

Stroke			
**CpG site**	**Control Subjects (*n* = 10)**	**Disease Subjects (*n* = 47)**	***P* Value**
1 (HIF binding site)	12.1 (5.7)	4.8 (5.7)	**0.003**
2	6.0 (4.8)	3.6 (3.3)	0.148
3	10.9 (3.5)	9.6 (5.3)	0.368
4	2.9 (1.0)	2.6 (2.6)	0.473
5	6.2 (1.6)	4.1 (3.7)	**0.006**
6	8.9 (3.4)	3.6 (3.9)	**0.006**
7	8.1 (2.5)	5.0 (3.3)	**0.048**
8	3.4 (1.2)	1.9 (2.0)	**0.003**
9	7.8 (2.5)	5.0 (3.3)	**0.010**
10	2.1 (0.3)	1.5 (1.2)	**0.004**
11	2.0 (0.3)	1.9 (1.2)	0.361
12	3.0 (1.0)	1.6 (1.5)	**0.003**
13	3.9 (1.2)	2.3 (1.8)	**0.003**
14	3.1 (0.7)	2.1 (2.2)	**0.018**
15	5.5 (1.3)	3.4 (3.2)	**0.002**
16	3.5 (1.2)	1.9 (1.9)	**0.002**
17	21.5 (7.7)	13.9 (14.5)	**0.026**
18	2.6 (0.7)	2.4 (2.3)	0.540
Average	6.3 (4.9)	4.0 (3.2)	0.100

Significant ($P < 0.05$) values are in bold.

Supplementary Table S3. DNA methylation changes of miR-210 gene promoter from non early relapse patients comparing with early relapse subjects in Colorectal cancer

Colorectal cancer			
**CpG site**	**Non early relapse (*n* = 30)**	**Early relapse (*n* = 8)**	***P* Value**
1 (HIF binding site)	6.0 (4.7)	2.5 (2.8)	**0.017**
2	4.2 (2.9)	3.7 (2.4)	0.576
3	8.8 (4.3)	7.9 (3.4)	0.537
4	2.5 (1.6)	2.8 (1.7)	0.710
5	4.4 (2.4)	4.3 (2.6)	0.959
6	5.5 (3.9)	3.8 (2.5)	0.152
7	6.0 (3.5)	5.6 (2.5)	0.668
8	2.4 (1.3)	1.9 (1.7)	0.453
9	5.2 (2.1)	5.6 (1.5)	0.520
10	1.8 (0.8)	1.7 (1.2)	0.902
11	2.2 (0.9)	2.9 (0.9)	0.092
12	2.1 (1.4)	1.1 (1.1)	**0.044**
13	2.4 (1.2)	2.2 (1.4)	0.703
14	2.3 (1.6)	0.8 (1.3)	**0.015**
15	4.5 (3.1)	5.6 (3.4)	0.424
16	3.0 (5.2)	1.6 (1.5)	0.187
17	10.0 (5.9)	6.8 (3.0)	**0.044**
18	1.9 (1.6)	2.1 (1.8)	0.836
Average	4.2 (2.4)	3.5 (2.1)	0.354

Significant ($P < 0.05$) values are in bold.

Supplementary Table S4. DNA methylation changes of miR-210 gene promoter from non -recurrence patients comparing with recurrence subjects in Oral cancer

Oral cancer			
**CpG site**	**Non-recurrence (*n* = 11)**	**Recurrence (*n* = 10)**	***P* Value**
1 (HIF binding site)	4.4 (2.9)	2.1 (1.7)	**0.043**
2	2.9 (3.1)	2.9 (3.6)	0.990
3	5.4 (3.6)	5.9 (4.7)	0.769
4	1.3 (1.6)	1.7 (1.8)	0.644
5	4.0 (2.6)	3.3 (2.6)	0.561
6	5.2 (2.4)	2.8 (2.1)	**0.024**
7	3.4 (2.4)	3.4 (3.3)	0.971
8	2.4 (1.7)	0.8 (1.4)	**0.028**
9	4.3 (2.8)	6.0 (3.8)	0.258
10	2.5 (1.5)	1.0 (1.1)	**0.016**
11	1.3 (1.5)	2.3 (2.1)	0.230
12	0.8 (1.3)	0.3 (0.6)	0.278
13	1.9 (1.8)	2.3 (2.1)	0.637
14	0.5 (0.7)	1.9 (2.6)	0.123
15	2.9 (2.0)	2.4 (2.0)	0.619
16	1.2 (1.5)	2.0 (1.9)	0.277
17	11.3 (3.0)	6.6 (5.8)	**0.038**
18	2.2 (1.6)	2.6 (2.1)	0.596
Average	3.2 (2.5)	2.8 (1.8)	0.573

Significant ($P < 0.05$) values are in bold.

Supplementary Table S5. DNA methylation changes of miR-210 gene promoter from Stage II patients comparing with Stage III subjects in Gastric cancer

	Gastric cancer		
**CpG site**	**Stage II (*n* = 11)**	**Stage III (*n* = 9)**	***P* Value**
1 (HIF binding site)	8.4 (5.6)	2.0 (0.6)	**0.003**
2	5.0 (3.4)	2.1 (0.4)	**0.018**
3	8.5 (5.9)	4.0 (0.9)	**0.030**
4	2.9 (2.1)	1.4 (0.3)	**0.046**
5	4.1 (2.5)	2.5 (0.4)	0.062
6	6.1 (3.6)	3.3 (0.8)	**0.028**
7	5.6 (4.4)	2.9 (0.5)	0.075
8	2.6 (2.0)	1.3 (0.3)	0.065
9	5.1 (3.6)	3.0 (0.6)	0.090
10	1.5 (0.7)	1.4 (0.3)	0.466
11	2.3 (1.8)	1.4 (0.2)	0.139
12	2.5 (1.5)	1.3 (0.3)	**0.031**
13	2.6 (1.5)	1.4 (0.2)	**0.023**
14	2.9 (2.3)	1.3 (0.3)	0.058
15	4.4 (4.2)	2.1 (0.6)	0.102
16	1.5 (1.1)	1.0 (0.4)	0.199
17	6.9 (3.1)	5.6 (1.5)	0.226
18	2.0 (1.1)	1.2 (0.5)	0.053
Average	4.2 (2.2)	2.2 (1.2)	**0.003**

Significant ($P < 0.05$) values are in bold.

Supplementary Table S6. PCR Primer

Primer	Sequence
**For cloning full-length HIF-1α**	
HIF-clone-F	ATTGAGCTCATGGAGGGCGCCGGCGGC
HIF-clone-R	GACCGGATCCTCAGTTAACTTGATCCAAAGCTCTG
**For cloning full-length SPRED2**	
SPRED2-clone-F	ACGGAATTCATGACCGAAGAAACACACCCAGAC
SPRED2-clone-R	ATTGGATCCTCACGCGGCCGCTTTGTGC
**For cloning SPRED2 3'UTR**	
SPRED2-3UTR-F1	TAAACGCGTTCAGTTTCCCTCCCTTCTCCC
SPRED2-3UTR-R1	TAAAAGCTTCTGATAGGATGTGTTTATATT
For cloning SPRED2 mutant 3'UTR	
SPRED2-3UTR-MU-F	TTATTTCAATAAGAAC**CGCGG**TATCCCAGCTGTTTT
SPRED2-3UTR-MU-R	AAAACAGCTGGGATA**CCGCG**GTTCTTATTGAAATAA
**For cloning miR-210 promoter**	
miR210-promoter-F	ATTCTCGAGGGCGGGAGGAGGACCACCTC
miR210-promoter-R	AATAAGCTTGGGCGGGCGGAGGGATTGAC
**For cloning miR-210-truncated promoter**	
miR-210-prom-del-F	GCCGCCCCTCCCCGGGCACAGAAAAGAACGCGCCGG
miR-210-prom-del-R	CCGGCGCGTTCTTTTCTGTGCCCGGGGAGGGGCGGC
**For BSP assay (human)**	
miR-210-M1-F	GGGTCGTTAGGTAAATTAGGTAGA
miR-210-M1-R	CGAAAACTACCCCTCTTCC
miR-210-M2-F	GGCGGGAGGAGGATTATT
miR-210-M2-R	CCTAATCCCTCAACCAATAACC
miR-210-M3-F	GTTGAAGTTGGGTCGAGAGT
miR-210-M3-R	CCCAAACACAAATCAACC
For BSP assay (mouse)	
Mou-miR210-BSP-F	AGGAAAAGGTATGTTTTGGGT
Mou-miR210-BSP-R	AAACCCACCCTAAAAAACTACA
**For MSP assay**	
210MSP-Me-F	TCGGGTCGTATTATTCGTTC
210MSP-Me-R	TCTTTTCTACACGTCTACCCG
210MSP-UM-F	ATTTTGGGTTGTATTATTTGTTT
210MSP-UM-R	TCTTTTCTACACATCTACCCAAAA
**For pyrosequencing assay**	
Forward primer	GGGAAGAGGGGTAGTTTT
Reverse primer	biotin-ACAAATAACCTAATCCCTCAAC

(*continued*)

Primer	Sequence
Sequencing primer	GAGTTAGGGTTATATT
**For ChIP assay**	
miR210chip-F	GGCAGACGTGCAGAAAAGA
miR210chip-R	CTGGTCCCTCAGCCAATG
**For quantitative real time PCR (human)**	
SPRED2-sybr-F	TATATTGTGCGTGTCAAGGCTG
SPRED2-sybr-R	GGGGTGCATGACCTTACAGA
SPRED1-sybr-F	AAGGATGCCCCGAATCAAAAA
SPRED1-sybr-R	GGCTTGGCTTTGCATGTAGAC
SPRED3-sybr-F	TCCTCCTCCTCCTCTCCTTC
SPRED3-sybr-R	CCTGAAGCTGACTCCATCGT
HIF1A sybr F	CCAGCAGACTCAAATACAAGAACC
HIF1A sybr R	TGTATGTGGGTAGGAGATGGAGAT
**For quantitative real time PCR (mouse)**	
M-spred2-sybr-F	AGGGAGCAGGGTGTTACTACT
M-spred2-sybr-R	ACTGAGCTTGATCTCTACCGT
